# Pulsed Feedback Defers Cellular Differentiation

**DOI:** 10.1371/journal.pbio.1001252

**Published:** 2012-01-31

**Authors:** Joe H. Levine, Michelle E. Fontes, Jonathan Dworkin, Michael B. Elowitz

**Affiliations:** 1 Howard Hughes Medical Institute and Division of Biology and Department of Applied Physics, California Institute of Technology, Pasadena, California, United States of America; 2 Department of Microbiology, College of Physicians and Surgeons, Columbia University, New York, New York, United States of America; Massachusetts Institute of Technology, United States of America

## Abstract

Environmental signals induce diverse cellular differentiation programs. In certain systems, cells defer differentiation for extended time periods after the signal appears, proliferating through multiple rounds of cell division before committing to a new fate. How can cells set a deferral time much longer than the cell cycle? Here we study *Bacillus subtilis* cells that respond to sudden nutrient limitation with multiple rounds of growth and division before differentiating into spores. A well-characterized genetic circuit controls the concentration and phosphorylation of the master regulator Spo0A, which rises to a critical concentration to initiate sporulation. However, it remains unclear how this circuit enables cells to defer sporulation for multiple cell cycles. Using quantitative time-lapse fluorescence microscopy of Spo0A dynamics in individual cells, we observed pulses of Spo0A phosphorylation at a characteristic cell cycle phase. Pulse amplitudes grew systematically and cell-autonomously over multiple cell cycles leading up to sporulation. This pulse growth required a key positive feedback loop involving the sporulation kinases, without which the deferral of sporulation became ultrasensitive to kinase expression. Thus, deferral is controlled by a pulsed positive feedback loop in which kinase expression is activated by pulses of Spo0A phosphorylation. This pulsed positive feedback architecture provides a more robust mechanism for setting deferral times than constitutive kinase expression. Finally, using mathematical modeling, we show how pulsing and time delays together enable “polyphasic” positive feedback, in which different parts of a feedback loop are active at different times. Polyphasic feedback can enable more accurate tuning of long deferral times. Together, these results suggest that *Bacillus subtilis* uses a pulsed positive feedback loop to implement a “timer” that operates over timescales much longer than a cell cycle.

## Abstract 2

In response to sudden environmental stress, *B. subtilis* cells can defer sporulation for multiple cell cycles using a pulsed positive feedback loop.

## Author Summary

How long should a cell wait to respond to an environmental change? While many pathways such as those affecting chemotaxis respond to environmental signals quickly, in other contexts a cell may want to defer its response until long after the signal's onset—sometimes waiting multiple cell cycles. How can cells create “timers” to regulate these long deferrals? We study this question in the bacterium *Bacillus subtilis*, which responds to stress by transforming into a dormant spore. We show that *B. subtilis* can defer sporulation for extended time periods by first undergoing multiple rounds of growth and proliferation, and only then sporulating. The timer for this deferral is a pulsed positive feedback loop, which ratchets up the concentration of the sporulation master-regulator Spo0A to a critical level over multiple cell cycles. Finally, using mathematical modeling, we illustrate how a novel dynamic feedback mechanism, “polyphasic positive feedback,” lets cells defer sporulation more robustly than with other circuit strategies. Developing techniques that can access pulsing and time-delay dynamics with higher time resolution will enable us to determine if this polyphasic strategy provides a general design principle for the regulation of multi-cell-cycle deferral times seen in other systems.

## Introduction

Cells are capable of responding to stimuli extremely rapidly, on timescales of seconds or less [1]. In some situations, however, cells respond to stimuli only after extended delays of multiple cell cycles. A classic example occurs in the developing mammalian nervous system, where, in the presence of appropriate signaling molecules, precursor cells will proliferate for up to eight cell generations before differentiating into oligodendrocytes [2]. Although many aspects of the system remain unclear, oligodendrocyte differentiation is similarly delayed in vivo and in cell culture, suggesting a cell-autonomous “timer” mechanism. Another example is the mid-blastula transition in developing Xenopus embryos, which occurs after 12 cell cycles of proliferation [3],[4]. In both cases, the deferral of differentiation enables a period of proliferation preceding commitment to new fates.

In bacteria, non-cell-autonomous strategies for deferring responses are well known. For example, in the marine bioluminescent bacterium *Vibrio fischeri*, cells use quorum sensing mechanisms to defer light production until the population reaches a critical density [5]. Similarly, *Bacillus subtilis* can defer sporulation through cannibalism [6],[7], a response triggered by cell-cell signaling at high cell density, in which one subpopulation of cells lyses another, releasing nutrients that sustain growth.

Although there has been much work on circuit architectures that speed response times [8], fewer studies have addressed cell-autonomous deferral mechanisms. Cell autonomous deferral requires the cell to keep track of the total time or number of division events since the appearance of the stimulus. It has remained unclear whether and how individual bacterial cells can achieve this functionality using genetic circuit components. The key problem is that as the cell grows and divides, its components dilute out. This dilution process sets an effective upper limit to the typical timescale over which the concentration of a protein responds to a step change in its production rate [8]. For example, a step change in the rate of production of a stable protein causes the concentration of that protein to exponentially approach its new steady-state value with a timescale of one cell cycle [9]. Thus, most gene circuits tend to relax to new steady states over timescales close to, or faster than, that of the cell cycle. Alternatively, genetic circuits can give rise to long deferral times in some cells through occasional stochastic switching between metastable states. While such systems can be tuned to generate long mean intervals between switching events, without cascades of multiple states, these mechanisms cannot generate well-defined, unimodal distributions of deferral times across a population [10],[11].

**Figure 1:**
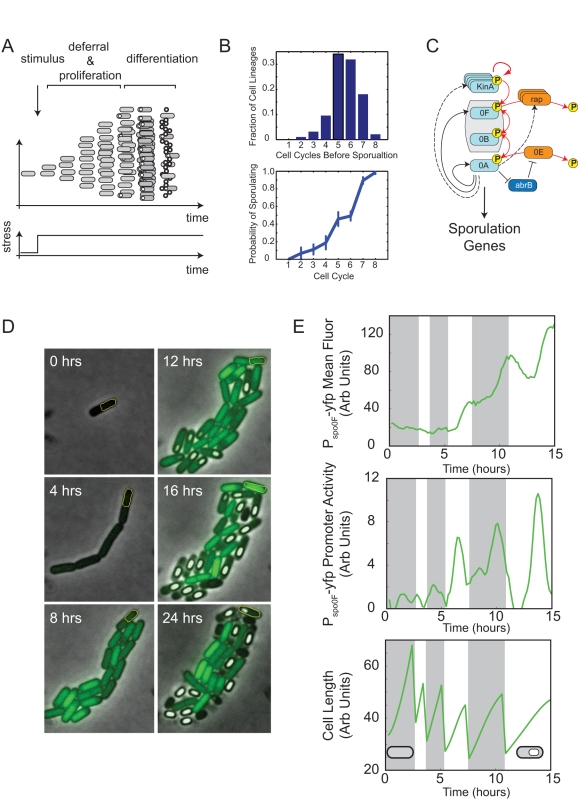
Pulsed Spo0A activity dynamics occur during the deferral of sporulation initiation in *B. subtilis*. (A) In some conditions, in response to sudden nutrient limitation, cells first proliferate for multiple cell cycles and then initiate differentiation into spores. (B) Top: Distribution of number of cell cycles from nutrient limitation to sporulation, from five different movies taken on two different days. Bottom: Probability of sporulating for each cell cycle following nutrient limitation, defined as the fraction of cells at that round of division which sporulate instead of continuing growth. Error bars are standard error of measurement of the probability computed across the five movies. (C) Phosphorelay gene circuit controlling sporulation initiation (abridged). Kinases, including KinA, autophosphorylate and transfer phosphates to the master regulator Spo0A (denoted 0A) via Spo0F and Spo0B (red arrows). Phosphatases (orange) and transcriptional regulators (blue) also impact the system response. Dashed arrows indicate indirect transcriptional regulation by Spo0A^P^. (D) Typical filmstrip showing growth and sporulation in a *B. subtilis* microcolony. Cells contain a P*_spo0F_*-*yfp* reporter construct (green). Note the multi-cell-cycle increase in fluorescence intensity. (E) Quantitation of YFP expression from the cell lineage outlined in yellow in (D). Mean fluorescence intensity (top) shows abrupt changes in fluorescence accumulation. The promoter activity (middle, see Materials and Methods), which is inferred from rates of change of fluorescence and cell size (bottom), reveals pulses in the rate of YFP production from the P*_spo0F_* promoter.

*B. subtilis* sporulation provides an ideal model system to address this problem. Sporulation is a canonical microbial stress response behavior, during which cells respond to stress by differentiating into an environmentally resistant spore. Sporulation is a terminal differentiation decision, and its initiation is regulated by a well-characterized genetic circuit whose dynamics can be analyzed in individual cell lineages [12]. This circuit, in response to diverse environmental and metabolic signals [13], controls the activation of the master regulator Spo0A through transcriptional regulation and phosphorylation [14]. High levels of phosphorylated Spo0A (Spo0A^P^) are sufficient to induce sporulation [15]. However, under some conditions, Spo0A^P^ levels increase gradually over multiple cell cycles, allowing cells to proliferate prior to differentiation. The ability to defer sporulation while proliferating could provide a fitness advantage to cells by increasing their numbers relative to immediate sporulators (Figure 1A), although it could also impose a cost to cells that do not sporulate in time to survive extreme conditions. During the deferral period, cells may also explore other fates, such as biofilm formation, which are known to occur at intermediate levels of Spo0A^P^ [16].

The genetic circuitry controlling Spo0A activation includes multiple types of interactions (Figure 1C). Histidine kinases such as KinA, KinB, and others autophosphorylate and transfer phosphates through a phosphorelay consisting of Spo0F and Spo0B to Spo0A [17]. Phosphatases reduce the total level of Spo0A^P^. For example, Spo0E directly dephosphorylates Spo0A^P^ [18], while *rap* phosphatases drain phosphates from the phosphorelay through Spo0F [19]. The system also includes extensive transcriptional regulation. Spo0A^P^ regulates its own transcription as well as that of *spo0F*. It also regulates many other genes, including global regulators such as AbrB [20]. Finally, Spo0A^P^ also indirectly regulates its own activity by activating kinase expression [21]. These transcriptional interactions typically occur at much longer timescales than the fast phosphotransfer reactions of the phosphorelay. Nevertheless, it remains unclear whether and how this circuit facilitates deferred differentiation.

Here, using time-lapse fluorescence microscopy of individual cells, we show that under some conditions *B. subtilis* cells defer sporulation for multiple cell cycles through a predominantly cell-autonomous mechanism. We observed a progressively increasing series of pulses of Spo0A phosphorylation during deferral. Manipulation of circuit interactions revealed that pulse growth and regulated deferral both required positive feedback on kinase expression. These results suggest that *B. subtilis* uses a pulsed positive feedback loop to gradually “ratchet up” Spo0A^P^ activity pulses over multiple cell cycles in order to defer sporulation. Finally, mathematical modeling of this mechanism further suggests that pulsing could enable a “polyphasic” feedback mechanism, in which different parts of the overall positive feedback loop are active at different times, facilitating regulation of deferral. This may be a general strategy that cells can use to enable regulation of timescales much longer than the cell cycle.

## Results

In order to analyze the sporulation initiation circuit in individual cells, we utilized a programmable time-lapse microscopy and quantitative image analysis system similar to those described previously [22],[23]. We grew cells in Casein Hydrolysate (CH) growth media [24] and then transferred them to agarose pads made with nutrient-limited media (RM) to induce sporulation during imaging [25]. Under these conditions, individual cells exhibited a peaked distribution of ∼5.5±1.3 (mean ± SD) rounds of cell growth and division before initiating sporulation (Figure 1B, top). The probability of sporulating each round of growth, defined as the fraction of cells sporulating that round, increased monotonically (Figure 1B, bottom). This suggests that cells individually defer sporulation for multiple cell cycles, and rules out alternative Poisson-like models in which sporulation occurs with a fixed probability per cell cycle.

### Pulsatile Activation of Spo0A

In order to understand how deferral is achieved, we set out to observe phosphorelay circuit dynamics during the deferral period. To read out Spo0A^P^ activity we chromosomally integrated a P*_spo0F_*-*yfp* reporter construct. The P*_spo0F_* promoter exhibits a high affinity for Spo0A^P^ and is therefore classified as a low-threshold activated gene [21]. To quantify P*_spo0F_* activity over time, we computed its YFP production rate (promoter activity) in single cells. Promoter activity takes into account measurements of the change in total cellular fluorescence between time-points, the instantaneous cellular growth rate (which varies considerably, even within a single cell lineage, Figure 1E, bottom panel), and other cellular parameters (Materials and Methods). Compared to mean cellular fluorescence, whose interpretation is complicated by the stability of fluorescent proteins, promoter activity better reflects production from P*_spo0F_* and thus Spo0A^P^ dynamics. We also inserted a constitutively expressed red fluorescent expression construct, P*_trpE_-mCherry*, which we used to aid in the automatic segmentation of cells in images.

P*_spo0F_* promoter activity could be observed in discrete pulses in individual cells, similar to those reported previously (Figure 1D,E) [26]. These pulses began after transfer to nutrient-limited conditions and continued until sporulation. In contrast, cells in rich media exhibited no measurable production from the P*_spo0F_* promoter, or sporulation associated genes generally. Pulses were not specific to the P*_spo0F_* reporter, but were observed across a range of Spo0A target genes (Figure S1), affecting many processes in the cell, including the expression of the global regulator *abrB* [27],[28] and *sdp*, a component of the “cannibalism” pathway [6],[21]. However, the phasing of pulses relative to the cell cycle differed between promoters, reflecting their different regulation modes (Figure S1). For example, Spo0A^P^-activated and Spo0A^P^-repressed promoters showed opposite phasing with respect to the cell cycle (Figure S1B). Each cell cycle typically contained one pulse (Figure S1C). Promoters *not* regulated by Spo0A, such as the σ^A^-dependent P*_trpE_* promoter, sometimes fluctuated in expression but exhibited a much smaller dynamic range, and no characteristic cell cycle phasing, and were thus qualitatively different from Spo0A-dependent pulsing (Figure S3).

In principle, pulses could be caused by a change in either the abundance or the phosphorylation state of Spo0A. To eliminate both transcriptional and phosphorylation control of Spo0A activity, we replaced *spo0A* with the well-characterized, constitutively active variant *spo0A^sad67^* [29], under the control of the IPTG-regulated hyperspank (P*_hyp_*) promoter. Although P*_spo0F_* was activated in response to *spo0A^sad67^* induction, this strain showed no pulsing (Figure S4), consistent with the fact that *spo0A^sad67^* does not require phosphorylation to be active. In addition, very few cells formed phase bright spores. The potential for pleiotropic effects of *spo0A^sad67^* expression prevents us from concluding that successful sporulation requires pulsing. On the other hand, a strain in which *spo0A* was under the control of P*_hyp_* retained similar pulse dynamics as wild-type (Figure S5) and consistently formed phase bright spores. These results strongly indicate that phosphorylation of wild type Spo0A is required for pulsation.

What molecular mechanism could be responsible for pulse generation? The sporulation kinase inhibitor Sda is regulated in a cell-cycle-dependent fashion, suggesting that it might be involved in pulse generation [30]. A null *sda* mutant exhibited increased mean Spo0A activity, and therefore strongly reduced the dynamic promoter activity of the sensitive Spo0A-repressed P*_abrB_* promoter [21], as observed previously (Figure S6) [26]. However, in the Δ*sda* mutant, P*_spo0F_* continued to pulse similarly to wild-type, showing that while Sda modulates the dynamic range of Spo0A activity, it is not required for pulsing. Intrinsic network dynamics involving negative feedback loops provide another possible pulse generation mechanism [31]–[33]. Together, *spo0A*, *abrB*, and *spo0E* form such a feedback loop (Figure 1B). However, deletion of *spo0E* did not eliminate pulsing (Figure S6). Other potential negative feedback loops involve Spo0A-dependent up-regulation of *rap* phosphatase expression. But deleting *rapA* and *rapB* individually and in combination similarly failed to abolish pulsing (Figure S6). Finally, we asked whether pulsing might be driven specifically by one of the phosphorelay kinases. In nutrient-limited conditions, KinA and KinB are the dominant phosphodonors [34],[35]. Strains lacking either *kinA* or *kinB* exhibited pulsed dynamics (Figure S7), suggesting that pulsation does not specifically require *kinA* or *kinB* individually. Together, these results show that pulsing is robust to deletion of a variety of different circuit components. While further elucidation of the mechanism of pulse generation will be important, we focus below on the consequences of pulsing for the deferral of sporulation.

### Deferral Time Is Regulated Cell-Autonomously by the Sporulation Initiation Circuit

**Figure 2:**
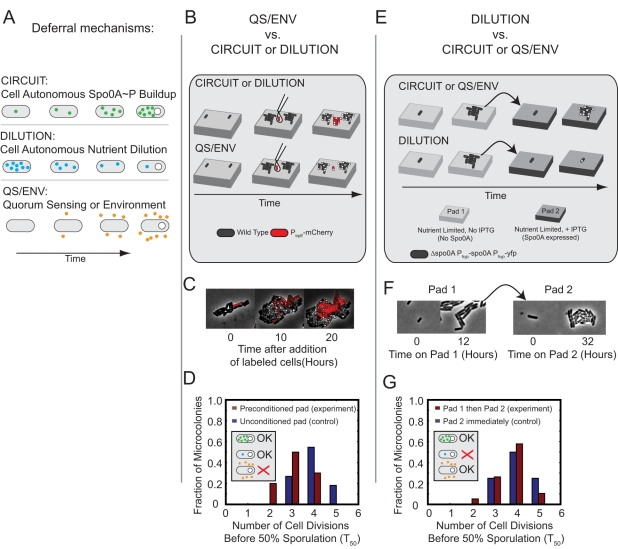
Multi-cell-cycle deferral is generated by cell-autonomous Spo0A circuit dynamics, rather than environmental changes or internal dilution. (A) Three classes of mechanism can generate deferred sporulation. Top (CIRCUIT): slow cell-autonomous circuit activation drives accumulation of Spo0A^P^ over multiple cell cycles. Middle (DILUTION): multiple cell divisions dilute out internal factors, such as nutrients, eventually permitting sporulation. Bottom (QS-ENV): slow changes in environment, possibly from environmental nutrient exhaustion or accumulation of extracellular quorum sensing signaling molecules, eventually permit sporulation. (B–D) Preconditioning agar pads with sporulating cells can test the QS/ENV mechanism. (B) Agarose pads are preconditioned by sporulating wild type cells (black). Fresh *mCherry*-labeled wild type cells (red) are subsequently added to conditioned pads once unlabeled cells near sporulation. If deferral is cell autonomous, the red cells will defer sporulation (top). If deferral depends on environment, red cells will sporulate immediately (bottom). (C) Filmstrip of labeled (red) cells placed on a pad preconditioned by unlabeled cells. (D) Sporulation time histograms (see Materials and Methods) for labeled cells placed on preconditioned resuspension media pads (red bars), compared with labeled cells on unconditioned resuspension media pads as a control (blue bars). (E–G) The pre-growth/pad transfer experiment can test the DILUTION mechanism. (E) Cells with an IPTG-inducible *spo0A* construct (P_hyp_-*spo0A*), but lacking the endogenous *spo0A* gene, are first grown for multiple generations on resuspension media pads without IPTG, diluting out nutrients or other factors present only in rich media. Cells are then transferred to a second resuspension media pad containing 100 µM IPTG. If deferral depends on the dilution of internal nutrients or factors, cells will sporulate immediately. If deferral depends on slow accumulation of a factor, cells will still defer sporulation. (F) Film strip of cells on pad 1 and pad 2. Note growth of individual cells into microcolonies on both pads. (G) Sporulation time histograms for cells placed first on pad 1, then transferred to pad 2 (red bars), compared to a control where cells are placed immediately on pad 2 (blue bars).

In principle, the extended multi-cell-cycle timescale for activation of Spo0A could be achieved in three different ways (Figure 2A): Internal genetic circuitry could generate a slow rise in a critical regulator (CIRCUIT cartoon). Alternatively, an inhibitor of sporulation could gradually dilute out over multiple cell cycles during the proliferation phase (DILUTION cartoon). Either of these two mechanisms would function cell-autonomously. Finally, cells could defer sporulation through a non-cell-autonomous mechanism involving the build-up of extracellular signaling molecules that modulate the phosphorelay (quorum sensing) [6],[36]–[39] or through degradation of the local micro-environment (QS/ENV cartoon). We performed two experiments that distinguish between these possibilities and, together, support a cell-autonomous mechanism that does not involve dilution for gradual build-up of Spo0A activity.

In the first experiment, we sought to distinguish between cell-autonomous and non-cell-autonomous deferral mechanisms. Low initial cell densities on resuspension media pads did not permit the growth and sporulation of cells, suggesting that at least some cell-generated factors were required for proliferation and possibly sporulation in these conditions. However, it was not clear whether these signals were responsible for deferring sporulation. To address this question, we developed a co-culture assay where unlabeled cells were mixed with red mCherry-labeled cells on the same pad (Figure 2B–D). The unlabeled wild-type cells were introduced ∼10 h before the labeled cells, allowing them to condition the pad as they proliferated and eventually sporulated (Figure 2B–D). If deferral were controlled by cell-extrinsic factors, then the red cells should sporulate earlier with the unlabeled cells than without them (Figure 2B lower cartoon). On the other hand, if the deferral of sporulation were cell-autonomous, then the red cells would proliferate for an equal amount of time in the presence or absence of the unlabeled cells (Figure 2B, upper cartoon).

In order to quantify this effect, we counted the number of cell cycles required for 50% of cells in a microcolony, starting from a single labeled cell, to initiate sporulation, as measured by the formation of a phase-bright forespore. Because the actual distribution of deferral times has a tail (Figure 1B), this measure, denoted as T_50_, approximates but slightly underestimates the actual mean deferral time as measured in individual cell lineages (Materials and Methods). We found that sporulation of labeled cells was only modestly accelerated by unlabeled sporulating microcolonies (Figure 2C and Figure 2D), reducing T_50_ by 25%, from ∼4 to ∼3 cell cycles. This measurement provides an upper limit to cell-extrinsic effects in our conditions. Although cell-extrinsic factors do play some role, deferral appears to be controlled in a predominantly cell-autonomous fashion.

We next sought to determine whether cell autonomous deferral in our conditions was caused by slow depletion of internal factors following the switch to resuspension media (Figure 2A, middle panel). One specific molecular candidate for the dilution mechanism is a slow depletion of intracellular GTP levels, which control repression of stationary phase genes by CodY through the alarmone (p)ppGpp [40]. However, in our experimental conditions, a Δ*codY* strain showed similar deferral behavior as wild-type cells (Figure S8), demonstrating that *codY* is not necessary for deferral.

Because the dilution mechanism need not work through *codY*, we designed an experiment to rule out the dilution model more generally (Figure 2E–G). In this experiment, a strain with IPTG-inducible *spo0A* (Δ*spo0A* P*_hyp_*-*spo0A*) is first grown on one nutrient-limited pad lacking IPTG and then transferred to a second, similar, nutrient-limited pad containing 100 µM IPTG (Figure 2E). The first pad allows cells to grow for multiple cell cycles without inducing sporulation (Figure S2A). This growth dilutes out putative internal factors not produced in nutrient-limited conditions. On the second pad, IPTG is present, enabling immediate constitutive transcription of Spo0A. After one cell cycle, Spo0A concentration reaches a steady state expression level at or exceeding that in sporulating wild-type cells (Figure S2B). In the dilution model, dilution of sporulation inhibitors during growth on the first pad would cause cells to sporulate immediately on the second pad. On the other hand, if deferral were due to cell-autonomous Spo0A circuit dynamics, growth on the first pad would have no effect on deferral on the second pad. In fact, the T_50_ distribution on the second pad was not substantially affected by 3–4 cell cycles of growth on Pad 1, with T_50_  =  4.1 ± 0.2 versus 4.4 ± 0.1 (mean ± SD) with and without Pad 1, respectively (Figure 2F,G). These results rule out the dilution-driven deferral model. Together, these results strongly suggest that multi-cell-cycle deferral is controlled by an extended cell-autonomous accumulation of Spo0A^P^.

### Kinase Levels Control the Deferral of Sporulation

To better understand how the sporulation initiation circuit controls deferral time, we consider two classes of genes. The first class consists of the phosphorelay genes Spo0A, Spo0F, and Spo0B and the sporulation kinases KinA–KinE, whose products directly contribute to the phosphorylation of Spo0A. Limited expression of these genes could potentially defer sporulation by slowing the phosphorylation of Spo0A. The second class consists of phosphorelay phosphatases, whose expression could potentially defer sporulation by draining phosphates from the relay, slowing the accumulation of phosphorylated Spo0A.

**Figure 3:**
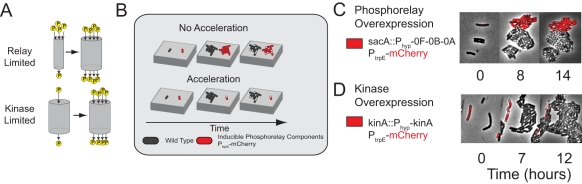
Spo0A^P^ activity growth is kinase-limited. (A) Two alternative regimes in which pulse growth could occur. (Left) The abundance of phosphorelay proteins is represented as the size of a “pipe” through which phosphates flow, allowing one to distinguish between a phosphorelay-limited regime (upper) where phosphorelay capacity increases with time, versus a kinase-limited regime (lower) where the flux of phosphates into the pipe limits Spo0A-phosphorylation. (B) Experimental scheme for distinguishing between the two regimes. *mCherry*-labeled cells (red) overexpressing a network component are compared to co-cultured wild type cells (black) grown in the same field of view on the same agarose pad (gray slab). Acceleration of sporulation, as shown, indicates that deferral time is limited by the overexpressed component(s). Co-culturing labeled and unlabeled cells together control for potential day-to-day and pad-to-pad variability. (C) Induced expression of an operon containing *spo0F*, *spo0B*, and *spo0A* fails to accelerate sporulation. (D) In contrast, induced expression of *kinA* accelerates sporulation.

We investigated the impact of these phosphorelay components on multi-cell-cycle deferral, distinguishing between two qualitatively different regimes, similar to an approach used previously (Figure 3A) [41]: In a *relay-limited* regime, phosphorelay protein concentrations (e.g. Spo0F and/or Spo0B and/or Spo0A itself) limit the rate of phosphotransfer and thus the level of Spo0A^P^. In contrast, in a *flux-limited* regime, the level of Spo0A^P^ is principally controlled by the rate at which phosphates are injected into the circuit by kinases and/or removed by phosphatases. To experimentally distinguish between the two regimes, we analyzed the behavior of unlabeled wild type cells alongside (cocultured with) *mCherry*-labeled cells engineered to overexpress different phosphorelay components. Overexpression of limiting components, but not non-limiting components, will accelerate sporulation relative to wild type as shown schematically in Figure 3B. Thus, overexpression of *spo0A* or an operon of phosphorelay components (*spo0A*, *spo0B*, and *spo0F*) should accelerate both Spo0A^P^ buildup and sporulation in the relay-limited regime, while having little to no effect in the flux-limited regime. Conversely, in the kinase-limited regime, kinase overexpression should accelerate both Spo0A^P^ buildup and sporulation in the flux-limited regime but have little or no effect in the relay-limited regime. We note that previous related work by Fujita and Losick has established the strong effects of *kinA* overexpression in a different context, showing that it is sufficient to induce immediate sporulation in rich media conditions, which strongly suppress sporulation altogether [15].

We observed little to no acceleration in the onset of sporulation when we expressed *spo0A* or the *spo0A-spo0B-spo0F* operon in the labeled cells, despite the ability of these constructs to complement corresponding mutants (Figure 3C). These cells sporulated with a T_50_  =  3.7 ± 0.2 (mean ± SD), similar to 4.0 ± 0.2 in wild-type cells. On the other hand, induced expression of *kinA* strongly accelerated both the activation of Spo0A, as measured by P*_spo0F_* expression, and the onset of sporulation (Figure 3D), resulting in T_50_  =  0.2 ± 0.1. These results suggest that the deferral of sporulation is flux-limited, but not relay-limited.

To further test whether kinases or phosphatases were responsible for flux limitation, we constructed strains lacking phosphorelay phosphatases individually and in combinations. Simultaneous deletion of *spo0E*, *rapA*, and *rapB* reduced deferral by about one cell cycle, but did not abolish the multi-cell-cycle deferral. Deletion of other phosphatase genes, including the Spo0A phosphatases *yisI* and *ynzD*, and the Spo0F phosphatases *rapE*, *rapH*, and *rapJ*, had no discernible effect (Figure S8). Evidently, phosphatases alone cannot explain the flux limitation underlying multi-cell-cycle deferral, whereas kinase over-expression is sufficient to abolish multi-cell-cycle deferral. Together, these results implicate the slow buildup of kinase as the predominant deferral mechanism.

This hypothesis is supported by analysis of a P*_kinA_*-*yfp* reporter, which confirmed that KinA concentration indeed builds up gradually in the cell cycles preceding sporulation, and does so to an extent that cannot be explained by the less than 2-fold slowing of growth rate during the experiment (Figure S9). Similarly, while cells on Pad 2 in the dilution experiment (Figure 2F) exhibited systematically slower growth rates than wild type cells (Figure S11), they still sporulated with a similar deferral period. Evidently, regulation of *kinA* expression leads to a progressive increase over multiple cell cycles.

### A Pulsed Positive Feedback Loop Controls Kinase Activity

One of the most prominent activators of the principle sporulation kinases *kinA* and *kinB* is Spo0A itself. Spo0A^P^ inhibition of the transcriptional repressor AbrB leads to up-regulation of *kinA* through σ^H^ [42] and de-repression of *kinB* [43]. Thus, increased kinase activity could be driven by the engagement of a positive feedback loop, in which Spo0A activity pulses activate kinase transcription, increasing the amplitude of subsequent Spo0A pulses, and thus ratcheting up kinase levels once per cell cycle. A comparison of Spo0A^P^ levels (inferred from P*_spo0F_* promoter activity) with KinA levels (measured with P*_kinA_*-*yfp* fluorescence) demonstrated that *kinA* expression correlates with Spo0A^P^ pulse amplitudes (Figure S9C). Imaging of a *kinA*-*gfp* protein fusion confirmed that KinA protein levels increase during the deferral period (Figure S10A) [44]. Furthermore, ectopic expression of a constitutively active *spo0A* mutant, *spo0A^sad67^*, in a *Δspo0A* background, led to full up-regulation of a P*_kinA_*-*yfp* reporter (Figure S10B), and no reporter expression was observed in this strain without induction of *spo0A^sad67^*. Together, these results indicate that active *spo0A* is necessary and sufficient for full *kinA* expression.

**Figure 4:**
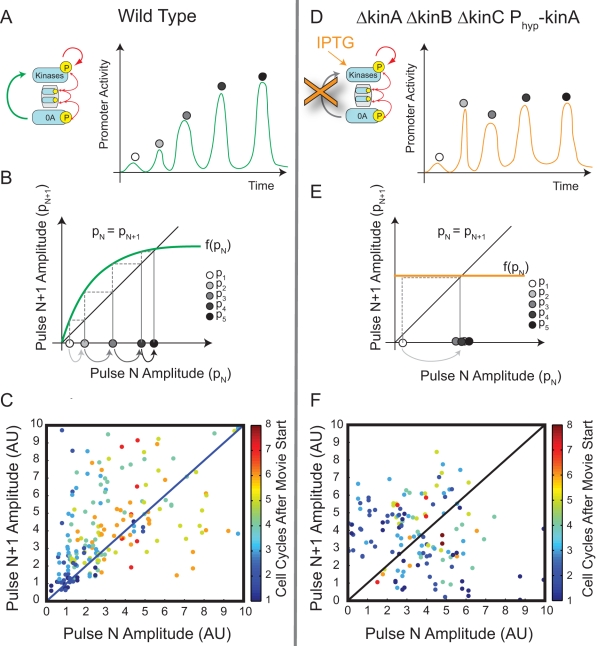
Return maps reveal that pulse growth depends on kinase feedback. (A) Pulse dynamics can be analyzed as a sequence of discrete pulse events. A schematic example, showing pulse growth in a hypothetical trajectory for the wild-type circuit (inset), is shown here (green line). Gray circles label successive peak amplitudes. (B) Schematic return map shows the progression of pulse sizes expected assuming a saturating positive feedback (green line). Here, a relatively weak initial pulse (white disk) leads to a larger value for the next pulse, whose value is determined by the feedback function (green line). When the feedback function intersects the diagonal line, successive pulses are equal in size and growth stops. Note that several pulses are required to reach the steady-state. (C) The experimental return map for wild-type cells. 247 individual pairs of successive pulses are plotted as dots. 165 pairs show growth and hence lie above the diagonal, while 82 pairs lie below the diagonal. (D–F) The feedback bypass strain (inset in D) shows qualitatively different dynamics. (D) Schematic showing expected behavior of this strain upon transfer to IPTG-containing media. Pulses should rapidly reach steady-state and not grow systematically with time due to the absence of positive feedback. (E) Return map (schematic) showing the progression of pulse sizes expected in a deterministic system lacking feedback, resulting in a “flat” feedback function (orange line). Note that a single step is sufficient to bring the system to steady-state (gray arrow). (F) The experimental return map for feedback bypass cells shows variability but less systematic pulse growth. 80 pulse pairs lie above the diagonal, while 64 pairs lie below the diagonal.

To investigate the potential role of this positive feedback loop, we developed a method to quantify pulse growth in individual cells. First, we characterized each P*_spo0F_* promoter activity pulse by its peak amplitude (Figure 4A). This allows promoter activity time traces to be represented by a discrete sequence of pulse amplitudes, one per cell cycle. We then plotted these pulse sequences on a “return map,” where the amplitude of each pulse (labeled p_N+1_) is plotted against the amplitude of the pulse immediately preceding it (labeled p_N_) (Figure 4B). Pulse growth causes points on the return map to lie above the diagonal line p_N_ = p_N+1_. In wild type cells, pulse amplitudes, though variable, tended to grow with successive cell cycles. Thus, on the return map, over two-thirds of data points lie above the diagonal, with the strongest growth at low and intermediate pulse amplitudes (Figure 4C). At high pulse amplitudes the trend saturates, so that the amplitude of a pulse eventually becomes independent of its predecessor. These results are consistent with the existence of a saturating positive feedback on kinase expression.

By contrast, if kinase expression were constitutive (Figure 4D), then induced kinase expression, and thus Spo0A^P^ pulse amplitude, would relax to a steady state with a timescale of about one cell cycle (similar to Figure S2B), eliminating systematic pulse growth (Figure 4E). To test this prediction experimentally, we constructed a “feedback bypass” strain, combining a Δ*kinA* Δ*kinB* Δ*kinC* triple deletion with IPTG-inducible *kinA* expression. In this strain, modest levels of IPTG (2 µM) allowed cells to grow and divide multiple times while activating Spo0A. Like wild-type cells, these cells exhibited variable amplitude Spo0A^P^ pulses correlated with the cell cycle (Figure S7). However, lacking transcriptional feedback on kinase expression, the pulse amplitudes showed no systematic growth over successive cell cycles (Figure 4F). These results suggest that feedback on kinase transcription is required for pulse growth.

**Figure 5:**
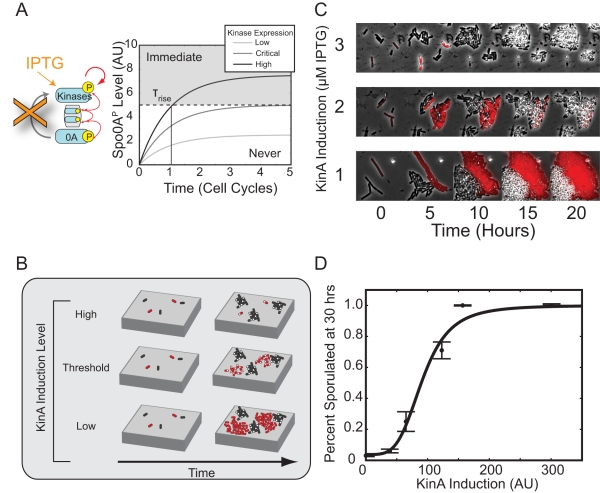
Positive feedback is required for control of rise time. (A) The deferral time is expected to be ultrasensitive to kinase induction levels in the absence of positive feedback. Excess kinase production causes Spo0A^P^ levels to rise and rapidly cross a sporulation threshold (dashed line), leading to immediate sporulation (gray region). By contrast, lower kinase production rates never produce enough Spo0A^P^ to cross the threshold (white region). Only an intermediate range of tightly tuned expression levels can potentially cross the threshold after significant delay (Critical). (B) Schematic of co-culture assay for comparing the proliferation of Δ*kinA* Δ*kinB* Δ*kinC* P*_hyp_*-*kinA* cells (red) with wild-type controls (black). Note the transition from rapid sporulation (small sporulating red microcolonies, top) to no sporulation (growing red non-sporulating microcolonies) as KinA induction level is decreased. (C) Sporulation timing depends sensitively on the level of *kinA* induction. Over a small change in IPTG level, we observed a sharp change in sporulation timing in Feedback Bypass cells (red). Note that no such change was observed among wild-type (black) cells. (D) Analysis of movies like those in (C) shows that the percentage of cells sporulating is a sharp function of IPTG as measured by YFP fluorescence from a P*_hyp_*-*yfp* construct and fit to a Hill function with Hill Coefficient *n*  =  4.0 ± 1.2 (95% confidence intervals). *y*-axis error bars are standard error of the mean.

Furthermore, the lack of pulse growth in the feedback bypass strain predicts an extremely sensitive dependence of sporulation timing on kinase expression levels. In this strain, IPTG concentration controls the steady-state kinase concentration, but not the timescale to reach it, which is set by the cell division time (Figure 5A). At low IPTG levels, Spo0A^P^ can never grow high enough to induce sporulation. Conversely, at high IPTG levels, sporulation would be induced almost immediately. Between these two extremes, sporulation would be deferred for multiple cell cycles only in a narrow window of kinase expression levels.

To test this prediction, we compared sporulation timing in our feedback bypass cells, labeled with mCherry, to that of wild-type cells co-cultured on the same agarose pad (Figure 5B). At low IPTG induction, these cells largely failed to sporulate, while at high induction levels, cells sporulated within one or two cell cycles (Figure 5C). The fraction of sporulated cells at 30 h showed a sharp dependence on *kinA* induction level, equivalent to a Hill coefficient of 4.1 ± 1.8 (95% confidence interval) (Figure 5D). Together, these results show that positive feedback on kinase expression is necessary for regulated deferral.

### Pulsed Positive Feedback as a Mechanism for Deferred Differentiation

How does positive feedback enable cells to set long deferral times, and what role can the pulsatile activation of Spo0A play? To explore these questions we constructed a mathematical model of the sporulation initiation circuit. We used a simplified model (Text S1) in order to gain insights into qualitative differences between different circuit architectures, but not to reproduce all known molecular interactions in the circuit. We modeled pulsatile Spo0A phosphorylation by activating kinase autophosphorylation for a fixed fraction of each cell cycle. We also simplified the phosphorelay into a two-component phosphotransfer from kinase to Spo0A. Although they are likely to be important for some aspects of the natural system, inclusion of Spo0F and Spo0B does not qualitatively affect the conclusions below. Finally, based on the insensitivity of deferral time to phosphatase deletions, we modeled phosphatase activity with a constant level of Spo0E.

Sporulation initiation is believed to require a threshold level of phosphorylated Spo0A. Indeed, we found that maximal P*_spo0F_*-*yfp* promoter activity in sporulating cells was systematically higher than in vegetative cells (Figure S12). Recently published experiments in bulk cultures have also demonstrated that cells sporulate at a threshold level of KinA [45]. Therefore, to analyze deferral, we quantified the number of cell cycles required for phosphorylated Spo0A to grow from a low initial level to a high threshold level.

**Figure 6:**
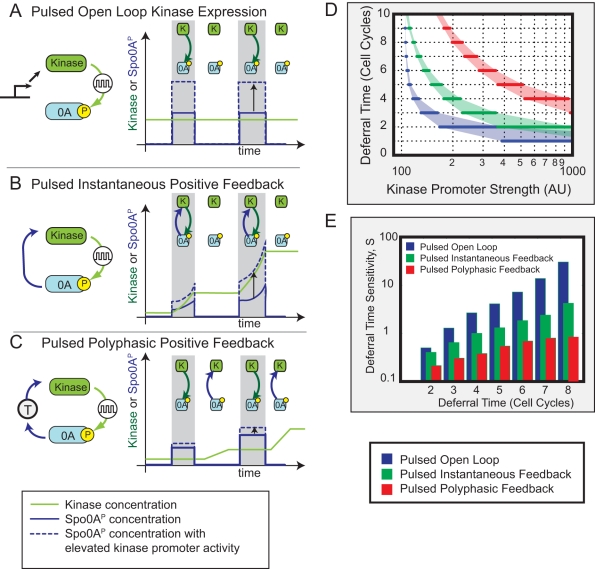
Pulsed polyphasic feedback can enable long deferral times with reduced sensitivity to parameters (schematic). Here we compare the ability of three simplified models of pulsed Spo0A^P^ regulation to generate extended, but tunable, deferral times. (A) In the pulsed open loop circuit, kinase activates Spo0A in pulses (gray regions), but there is no feedback. As a result, kinase levels are constant (green line), and changes in kinase expression level directly affect pulse amplitude, as shown by the dashed, compared to the solid, blue line. (B) In the pulsed instantaneous feedback circuit, increases in Spo0A^P^ cause immediate increases in kinase expression, reflected in the accelerated growth of kinase and Spo0A^P^ expression during, but not between, pulses. Thus, both arms of the feedback loop are active during, but not between, pulses (cartoon inserts). This results in strong sensitivity to kinase expression level and other parameters (compare solid and dashed blue lines). (C) In the polyphasic circuit, a time delay in the feedback loop causes periods of kinase accumulation to predominantly occur between, rather than during, Spo0A^P^ pulses. Effectively, the two arms of the feedback loop are active at different times (cartoon insets). As a result, an increase in kinase expression level has a more modest effect on pulse growth (dashed versus solid blue line). (D) Mathematical models of the three circuits were compared for their ability to generate multi-cell-cycle deferral times. For each circuit, the kinase promoter strength was tuned to produce different deferral times (*x*-axis). The sensitivity of deferral time to kinase promoter strength was defined as 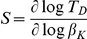, where *T_D_* is the deferral time in cell cycles, and *β_K_* is the strength of the kinase promoter. The three circuits differ systematically in both the magnitude and rate of increase of sensitivity with deferral time with the open loop circuit being most sensitive (cf. Figure 5), followed by the instantaneous and then the polyphasic circuit. Reduced sensitivity enables the cell to more accurately regulate deferral times. Note log *y*-axis scale, and see Text S1 for details.

We performed this analysis for three distinct circuit architectures, which differ in how kinase expression is controlled (Figure 6): In the first circuit, kinase is produced constitutively (open loop, Figure 6A). In the second, kinase production is instantaneously activated by Spo0A^P^ (pulsed instantaneous positive feedback, Figure 6B). In the third circuit, kinase is indirectly activated by Spo0A^P^, leading to an effective time delay (*τ*) between Spo0A phosphorylation and consequent up-regulation of kinase expression. If the Spo0A^P^ pulse terminates before kinase expression initiates, the pulsing and time delay together effectively divide the deferral period into distinct phases where either Spo0A^P^ pulsing or kinase transcription (or neither) is active, but never both; we call this type of feedback “polyphasic feedback” (Figure 6C).

In the polyphasic mechanism, τ could represent a number of possible intermediate processes including indirect regulation as well as transcriptional and translational time delays. Our current methods cannot firmly establish nor rule out such time delays, due to the relatively low time resolution inherent in promoter activity measurements made with fluorescent protein reporters. For example, we could not detect a consistent time difference between pulses of P*_kinA_*-*yfp* and P*_spo0F_*-*cfp* promoter activity (Figure S9B). Higher time resolution and methods to track protein phosphorylation in individual cells could help to constrain the exact magnitude of such effects.

For each circuit, we systematically modulated kinase or phosphatase production rates, both of which directly control phosphate flux to Spo0A. For each production rate, we monitored the time required for Spo0A^P^ to exceed a fixed threshold (deferral time, Figure 6D), and computed the sensitivity of this time to parameters such as the kinase production rate, 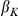 (Figure 6E). The open loop circuit showed an extremely sensitive dependence of deferral time on phosphorylation parameters (Figure 6D&E, blue), consistent with the sensitive dependence on kinase production observed experimentally (Figure 5D). The positive feedback loop reduced this sensitivity (Figure 6D&E, green), and the polyphasic feedback loop reduced it still further (Figure 6D&E, red). Models were tuned so the steady-state Spo0A^P^ levels in the polyphasic circuit exceeded those in the positive feedback circuit, ensuring that longer deferral times were not caused by lower steady states. Positive feedback, especially in the polyphasic regime, evidently could make it easier for cells to regulate multi-cell-cycle deferral times by reducing the sensitivity of deferral time to key control parameters [46].

How can we explain the relative sensitivities of the three circuits? In the open loop circuit, protein dilution due to cell growth determines the kinase concentration kinetics. The dilution rate is determined by the cell cycle time, making it difficult to achieve deferrals longer than a single cell cycle. In the positive feedback circuit, protein production and dilution compete with each other [47] to set the timescale of kinase accumulation. Parameters that affect net feedback strength (e.g., kinase or phosphatase promoter strengths) directly tune this timescale, and thereby modulate deferral time. Finally, the polyphasic positive feedback circuit includes the benefits of positive feedback. In addition, however, the combination of Spo0A^P^ pulsing and a time delay in its feedback onto kinase production together cause most of the new kinase produced by a pulse to appear only after the pulse terminates. Consequently, kinase cannot instantaneously feed back to amplify the pulse that produced it. Since feedback occurs from pulse to pulse, rather than compounding continuously as in standard positive feedback, pulse growth is much less sensitive to changes in feedback strength. Qualitative insights into the three circuit architectures can be obtained by analytically solving a set of corresponding simplified one-dimensional models (Text S1 and Figure S13). In these simplified models, although protein concentration grows exponentially in both positive feedback and polyphasic circuits, the time constant of the polyphasic circuit is exponentially less sensitive to feedback strength.

## Discussion

To respond properly to the challenges posed by environmental and developmental constraints, cells respond to stimuli across widely varying timescales. In some systems, the challenge is to achieve extremely rapid responses [1]. In other cases, however, cells may face the opposite challenge of deferring a response for relatively long times. Sporulation initiation represents an ideal example, where a sudden change in environment leads to a particular response—sporulation—only after many cell cycles. Although sporulation is deferred, it is clear that cells respond to the change in conditions throughout the deferral period, for example through continual increases in Spo0A activity.

In principle, several different mechanisms can produce a deferred response. Quorum sensing mechanisms can defer activation of a response until a critical cell density is reached, as occurs in the *V. harveyii* light production circuit [5]. Our data do not rule out a role for quorum sensing, but show that it cannot explain most of the multi-cell-cycle delay observed here (Figure 2B–D). Furthermore, since quorum sensing is a response to absolute cell density, rather than to rounds of cell division, it may be better suited to measuring population size as opposed to time intervals. A second potential mechanism for deferral is dilution of an internal molecule that represses sporulation. Dilution failed to explain sporulation deferral in our experiments (Figure 2E–G). A dilution mechanism requires the regulator to be produced continually before nutrient limitation at a level tuned to provide the appropriate deferral time during nutrient limitation. Thus, this strategy might be better adapted to a more deterministic environment, such as multicellular development [2], rather than the more unpredictable environments that microbes experience [48]–[50]. In contrast, the cell-autonomous feedback-dependent mechanism analyzed here allows deferral time to be quickly tuned from immediate to multiple cell cycles under different conditions. Indeed, previous studies of sporulation have used conditions optimized to induce immediate, rather than deferred, sporulation with the same circuit [24],[41]. The relative advantages of each type of mechanism may become clearer as additional examples of deferred differentiation are identified and elucidated.

Feedback loops are known to affect the response times of gene circuits. Negative feedback has been previously shown to accelerate responses [8]. Here we demonstrate the complementary role of positive feedback in extending timescales. This latter function is particularly important when proliferating cells need to postpone responses beyond a single cell cycle, the longest fundamental timescale of protein turnover in growing cells. Positive feedback extends timescales by competing with protein dilution to set the net relaxation time for protein concentrations. In *B. subtilis* sporulation, our results reduce the overall circuit to a core two-element positive feedback loop involving the master regulator Spo0A^P^ and the sporulation histidine kinase KinA. This feedback loop progressively ratchets up Spo0A^P^ levels, approaching the threshold level required for sporulation only after multiple cell cycles, and thereby enabling multi-cell-cycle deferral.

A striking aspect of the system analyzed here is pulsatile phosphorylation of Spo0A. Pulsing imposes additional temporal structure on circuit dynamics that can lead to novel regulatory capabilities. For example, the yeast transcription factor Crz1 undergoes discrete pulses of nuclear localization at a frequency set by extracellular calcium concentration [51]. The relative fraction of time Crz1 spends in and out of the nucleus is determined by the pulse frequency. This “FM” regulation sets the fraction of time that all of Crz1's targets are activated, leading to proportionally coordinated expression of the entire regulon.

In addition to quantizing responses, pulsing can also dictate the relative timings of different interactions in a circuit. Here, the pulsed buildup of Spo0A^P^ defers sporulation for multiple cell cycles through a Spo0A^P^-KinA positive feedback loop. When time delays are present in this feedback loop, increased KinA production occurs after the Spo0A^P^ pulse ends. As a result, Spo0A^P^ production and KinA production are temporally separated. In this “polyphasic” regime, pulsing and time delay work together to prevent instantaneous feedback, making the buildup rate significantly less sensitive to parameter values than it would be in a conventional positive feedback loop. It will be interesting to develop techniques that can access these dynamics with higher time resolution, and to see if this polyphasic strategy provides a general design principle for regulation of multi-cell-cycle deferral times in other systems.

Finally, one can ask whether deferring sporulation might have other benefits in addition to enabling proliferation. *B. subtilis* cells could explore alternative cell fates such as competence, biofilm formation, or cannibalism, during the deferral period. In this way, deferred progression to sporulation, implemented by a simple cell-autonomous pulsed positive feedback circuit, provides a critical foundation upon which multifaceted developmental programs can unfold.

## Materials and Methods

### Time Lapse Microscopy

#### Cell preparation

Overnight cultures grown to saturation in shaking CH (Casein Hydrolysate) media at 37°C with antibiotic selection were rediluted 1∶100 into 2.5 ml fresh CH media without selection. The resulting culture was then grown at 37°C to an OD_600_ of 0.8–1.0. Cells were then washed 2× and finally resuspended in the same final volume with fresh room temperature Resuspension Media (RM) [25]. 0.5 µl of resuspended cells was then spotted onto an appropriate agarose pad (see next paragraph). The cells and agarose pads were then covered, dried for approximately 15 min at room temperature, and then inverted into a glass coverslip bottom dish (Willco HBSt-5040), which was then parafilm sealed for imaging.

#### Agarose pad preparation

Agarose pads were prepared by melting 1.5% weight by volume low melting point agarose (OmniPur, EMD) into RM. 1 ml of liquid RM-agarose was then pipetted onto a 22 mm square coverslip, covered with a second coverslip, and allowed to dry covered at room temperature for at least 1 h. For experiments involving transgene induction, an appropriate amount of IPTG was added to cooled liquid RM-agarose and thoroughly mixed before pipetting onto the coverslip.

#### Microscopy

Cell growth and sporulation was observed at 37°C using an Olympus IX-81 inverted microscope controlled with custom software. Multiple stage positions were monitored using a motorized stage from ASI. Fluorescent reporters were excited by a 175 Watt Lambda LS Xenon arc lamp (Sutter), with typical exposure times between 200 ms and 1 s. Excitation light was filtered with a combination of neutral density and UV/IR filters to minimize phototoxicity and photobleaching. Images were recorded using an ORCA-ER camera (Hamamatsu) at a frame rate of once every 10 min, unless otherwise noted.

### Image and Data Analysis

Custom MATLAB software, similar to that described in Rosenfeld et al. [52], was used to extract time lapse fluorescence values for individual cells and lineages in a microcolony. All subsequent data analysis was also done in MATLAB using customized software.

#### Definition and extraction of promoter activity

We define promoter activity as the protein production rate from an individual allele of a promoter. To estimate this quantity from time lapse fluorescent data, consider reporter production dynamics from a promoter. In a given cell the total amount of fluorescent protein over time is denoted as *F*(*t*). The promoter activity *P*(*t*) represents the rate of production of *P*(*t*). Fluorescent protein is also degraded, diluted, and photobleached with a combined first-order rate constant γ. In our conditions, γ is dominated by dilution [23]. Thus, using a dot to denote the time derivative:
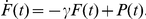
Using this relation we could determine *P*(*t*) from fluorescence time-series data by using a simple approximation for the derivative. For example, an Euler approach would yield the following expression:
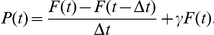
This type of explicit method, using a cell's total fluorescence, is sensitive to segmentation errors, such as the exact size and shape of the image region identified with the cell. We therefore derive an expression for promoter activity that uses only the observables of cell length and mean fluorescence, which we have found to be more robust to segmentation errors. First, we note that a cell's total fluorescence *F*(*t*) is its mean fluorescence *M*(*t*) multiplied by its area *A*(*t*). Since the widths of *Bacillus subtilis* cells remain essentially constant, we can write the following:
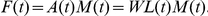
Here, *W* represents the constant cell width, and *F*(*t*) represents the time varying cell length. Inserting this into the definition of promoter activity, invoking the differentiation chain rule, neglecting the constant 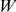 factor, and dividing by *L*(*t*), we are left with the following expression:
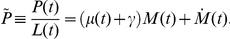
Here we have defined 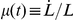 to be the cell's instantaneous growth rate. We have introduced 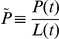 as an approximation to the production rate per chromosomal equivalent, facilitating comparison of production rate across all phases of the cell cycle. To understand the two terms on the right, consider two extreme limits. In the first limit, imagine the cell is growing but the mean fluorescence level remains unchanged, 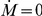. The first term is nonzero, however, implying that promoter activity results from production balancing out the effects of dilution. A second limit occurs when cell growth is negligible but mean fluorescence is increasing. In this case protein production is reflected in increasing protein concentration. In the main text, promoter activity refers to 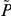, as defined above.

#### Characterization of promoter activity pulses

We statistically characterized promoter activity pulses using custom software routines written in MATLAB. We used a multi-step method to determine pulse locations in our time traces. Individual time traces were first filtered with a nonlinear smoothing method that replaces each point with the average of the points in a fixed window around it, after rejecting the highest point and the lowest point in the window. We used a window size of 7 (3 points on either side of center). We verified that our smoothing did not introduce any significant phase shift that would affect our temporal measurements. Potential pulses were then defined as local maxima in each smoothed trace (determined using a sliding boxcar method). For each putative pulse, we extracted its width (defined as the number of frames around the maximum with negative second derivative) and height, which we used to compute the pulse area. We set a minimum pulse size to prevent spurious identification of noise as pulses.

#### Definition of T_50_

To rapidly quantify typical sporulation times across multiple sporulating microcolonies, we use the T_50_ statistic described in the text. Briefly, in each microcolony we noted when 50% of cells exhibited a phase bright spore and then counted the total number of spores and non-spores. T_50_ is log_2_ of this number. T_50_ underestimates in two ways the mean number of generations for which cells defer sporulation. First, it can be shown that in a binary tree with *N* leaves, the average path length from a leaf to the root is lower bounded by log_2_(*N*). Second, because half the cells in each measurement have not sporulated, any additional growth and divisions by these cells will not be accounted for by T_50_.

## Supporting Information

Figure S1 Spo0A^P^ targets typically pulse once per cell cycle with defined phases. (A) Single cell time lapse traces of promoter activity in P*_abrB_*-cfp/P*_spo0F_*-yfp cells (JL013, top) and P*_sdp_*-cfp/P*_spo0F_*-yfp cells (JL072, bottom). Individual cell cycles are delineated by sequential gray and white shading. Cartoon indicates key regulatory links. (B) Left: illustration of definition of phase. Right: Histogram of cell cycle phases for *abrB* and *spo0F* promoter activity pulses. *abrB* expression typically pulses early each cell cycle, while spo0F expression typically pulses later. (C) Histogram of number of spo0F promoter activity pulses per cell cycle. Half of cells pulse in the first two cell cycles following transfer to resuspension media, while the majority of cells show a single pulse in subsequent cell cycles. The null hypothesis is a binomial distribution with the same mean number of pulses per cell cycle.
(PDF)

Figure S2 Analysis of pre-growth and Spo0A expression for pad transfer experiment (Figure 2E–G). (A) Distribution of number of cell divisions on Pad 1. The growth of 10 randomly chosen microcolonies were followed on Pad 1 using time lapse microscopy. (B) Induced Spo0A expression on Pad 2 (JL190, black—mean plus/minus SD) rapidly exceeds that from the wild type *spo0A* promoter (JL251, red—mean plus/minus SD). Fluorescence is mean cellular yfp intensity time averaged over the entire cell cycle.
(PDF)

Figure S3 The trpE promoter fluctuates but does not pulse. Typical time traces of P*_trpE_*-mCherry mean fluorescence (left) and promoter activity (center), along with cell length (right) in a typical cell lineage (strain JL024). Promoter activity, while fluctuating, has a lower dynamic range and less temporal structure than Spo0A^P^ regulated promoters.
(PDF)

Figure S4 Pulsing is abolished in the constitutively active Spo0A mutant Spo0A^sad67^. Typical time traces of P*_spo0F_*-yfp mean fluorescence (left) and promoter activity (center), along with cell length (right) in a typical cell lineage (strain JL065). The promoter activity exhibited fluctuations but lacked the characteristic cell cycle phased pulses present in the wild type.
(PDF)

Figure S5 The native spo0A promoter is not required for pulsing. Typical time traces of P*_spo0F_*-yfp mean fluorescence (left) and promoter activity (center), along with cell length (right) in a typical cell lineage of strain JL111 (Δspo0A P_hyperspank_-spo0A), showing pulsing similar to that observed in wild type cells.
(PDF)

Figure S6 Negative regulators of sporulation initiation are not required for pulsing. Typical time traces of P*_spo0F_*-yfp mean fluorescence (first row) and promoter activity (second row), P*_abrB_*-cfp mean fluorescence (third row) and promoter activity (fourth row), along with cell length (bottom row) in typical cell lineages of Dspo0E (strain JL014), Δsda (strain JL015), and ΔrapA ΔrapB (strain JL160). JL160 lacks the P*_abrB_*-cfp reporter present in the two other starins. Each strain exhibits Spo0A^P^ activity pulses in the P*_spo0F_* promoter similar to those seen in the wild type.
(PDF)

Figure S7 Major sporulation kinases are not individually required for pulsing. Typical time traces of P*_spo0F_*-yfp mean fluorescence (left) and promoter activity (center), along with cell length (right) in typical cell lineages of ΔkinA (strain JL090, top) and ΔkinAΔkinB P*_hyperspank_*-kinA (strain JL144, induced at 2 µM IPTG). Both strains exhibit clear pulsing in P*_spo0F_*-yfp promoter activity.
(PDF)

Figure S8 Negative regulators of sporulation initiation are not required for a multi-cell-cycle deferral. For each strain (left column), 10 sporulating colonies were tracked with time lapse microscopy and mean sporulation time in cell cycles quantified using the T_50_ statistic (right column). Despite some day-to-day variation, no strain ever exhibited mean sporulation time less than three cell cycles.
(PDF)

Figure S9 KinA levels increase gradually throughout the deferral period along with Spo0A^P^ pulse amplitudes. (A) Mean cellular fluorescence traces of cells (strain JL264) expressing P*_kinA_*-yfp and P*_spo0F_*-cfp. (B) P*_kinA_*-yfp mean fluorescence and P*_spo0F_*-cfp promoter activity in a single cell lineage. Alternating grey and white shading represents successive cell divisions. (C) P*_kinA_*-yfp mean cellular fluorescence correlates with Spo0A^P^ pulse amplitude. Each point represents a single cell's time averaged mean cellular yfp fluorescence (*x*-axis) and its maximum P*_spo0F_*-cfp promoter activity (*y*-axis). Point color represents that cell's depth in the lineage tree (cell cycles). Correlation coefficient *R*  =  0.68.
(PDF)

Figure S10 Regulation of Kinase Production. (A) kinA-gfp levels increase during sporulation. Mean cellular gfp fluorescence at movie start (left) and at sporulation (right). (B) Comparison of P*_kinA_*-yfp expression (mean cellular fluorescence) between cells with wild-type promoter regulation (left, BS264) and cells where P*_kinA_* is regulated by inducible spo0A^sad67^ (right, BS336). In wild type cells expression rises monotonically from movie start (blue) until sporulation (red). Fluorescence values in the inducible sad67 cell line were taken after 15 h on the resuspension media pad. Fluorescence in uninduced cells (blue) remained low, while the fluorescence of IPTG induced cells (red) was similar to that of sporulating wild type cells. Bars represent standard error of measurement.
(PDF)

Figure S11 Cell growth rate in pad transfer experiments. Histograms of cell growth rate, measured in cell cycle duration on pads 1 and 2 (cf. Figure 2E–G). Growth on Pad 2 (red, *N*  =  167) is significantly slower than growth on pad 1 (blue, *N*  =  105). Although Pad 2 cells grow significantly slower than wild type cells, they still defer sporulation for the same number of cell cycles (Figure 2G).
(PDF)

Figure S12 Sporulation occurs past a threshold level of Spo0A^P^. Histogram of maximal promoter activities (strain JL024) in our movie conditions. Non-sporulating cells (blue, *N*  =  109) showed systematically lower promoter activity than sporulating cells (red, *N*  =  55), although there is significant overlap. The conditional probability of sporulating given a promoter activity greater than 6 is 70%.
(PDF)

Figure S13 Simplified one-dimensional models capture qualitative circuit deferral behaviors. One-dimensional models are described in Text S1. (A) Dynamic traces of models tuned to cross a threshold of X  =  100, starting from X  =  1, with a five cell cycle deferral. X is plotted on both linear (left) and logarithmic (right) scales to illustrate exponential behavior. (B) Deferral time dependence on feedback strength for each model. For comparison, b of each model is plotted normalized to the minimal value b0 needed to reach the threshold of X  =  100. Open loop: b_0_  =  100; instantaneous: b_0_  =  1; polyphasic: b_0_  =  e−1. (C) One-dimensional models were compared for their ability to generate multi-cell-cycle deferral times, as with the two-component model of the main text. For each circuit, feedback strength b was tuned to produce different deferral times (*x*-axis). The sensitivity of deferral time to feedback strength was calculated as in the two-component model. The three circuits differ systematically in both the magnitude and rate of increase of sensitivity with deferral time. The open loop circuit is the most sensitive, followed by the instantaneous feedback, with the polyphasic feedback showing the least sensitivity.
(PDF)

Text S1 Details of experimental protocols (including strain construction) and mathematical modeling.
(DOC)

## Abbreviations

CH: Casein Hydrolysate
RM: Resuspension Media
Spo0A^P^: phosphorylated Spo0A
