# Supplementary figures and images for: Pulsed Feedback Defers Cellular Differentiation
Source: PLoS Biol. 2012 Jan 31;10(1):e1001252. doi: 10.1371/journal.pbio.1001252 (PMC3269414; doi:10.1371/journal.pbio.1001252)

# Figure S1

A

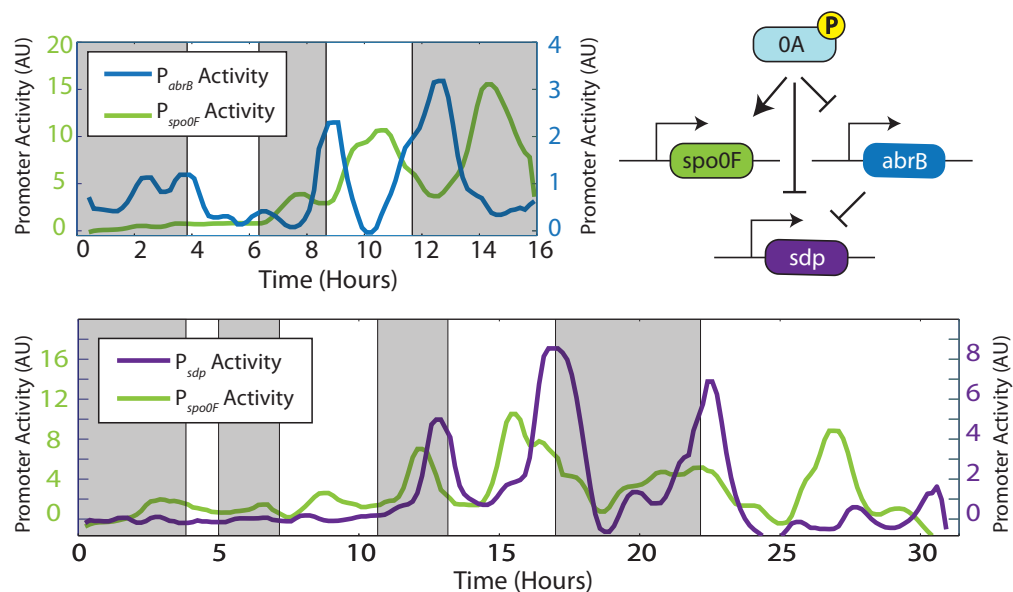

B

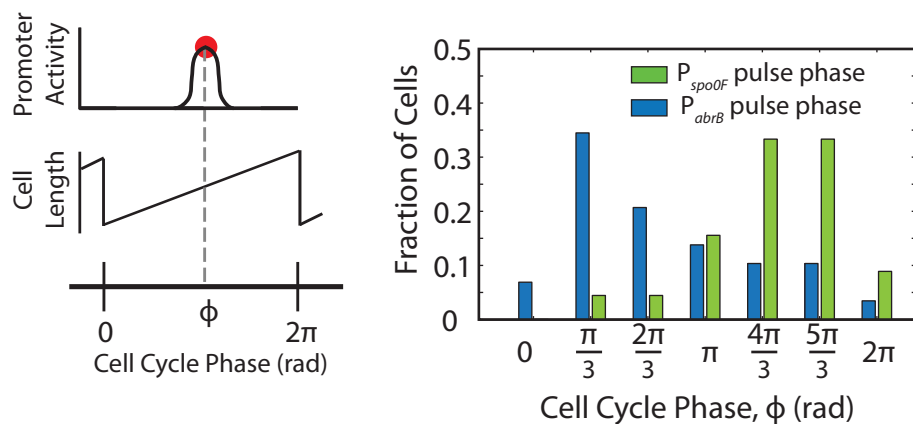

C

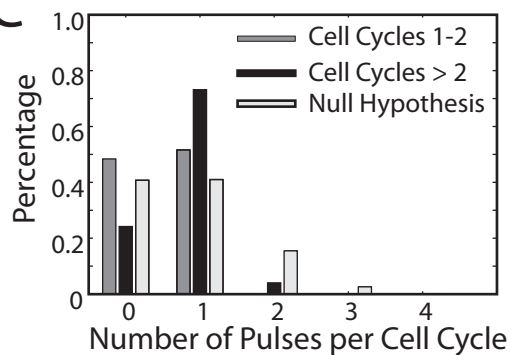

Supplement: Figure S1 — Spo0AP targets typically pulse once per cell cycle with defined phases. (A) Single cell time lapse traces of promoter activity in PabrB-cfp/Pspo0F-yfp cells (JL013, top) and Psdp-cfp/Pspo0F-yfp cells (JL072, bottom). Individual cell cycles are delineated by sequential gray and white shading. Cartoon indicates key regulatory links. (B) Left: illustration of definition of phase. Right: Histogram of cell cycle phases for abrB and spo0F promoter activity pulses. abrB expression typically pulses early each cell cycle, while spo0F expression typically pulses later. (C) Histogram of number of spo0F promoter activity pulses per cell cycle. Half of cells pulse in the first two cell cycles following transfer to resuspension media, while the majority of cells show a single pulse in subsequent cell cycles. The null hypothesis is a binomial distribution with the same mean number of pulses per cell cycle. (PDF) [file pbio.1001252.s001.pdf]

Figure S2

A

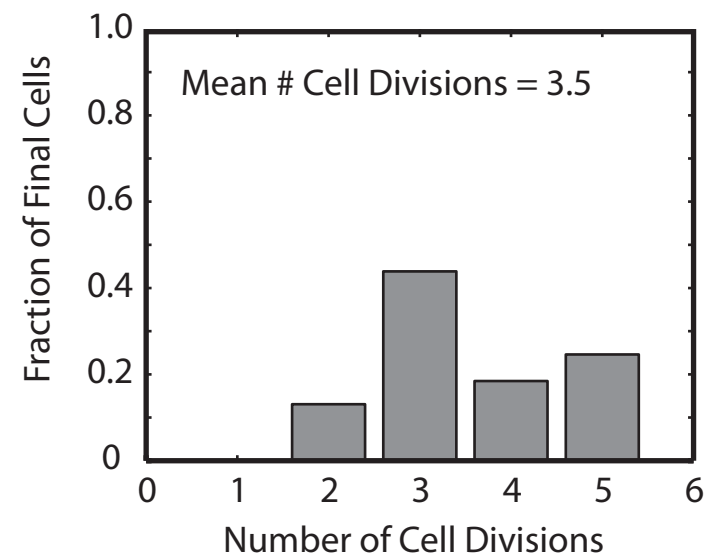

B

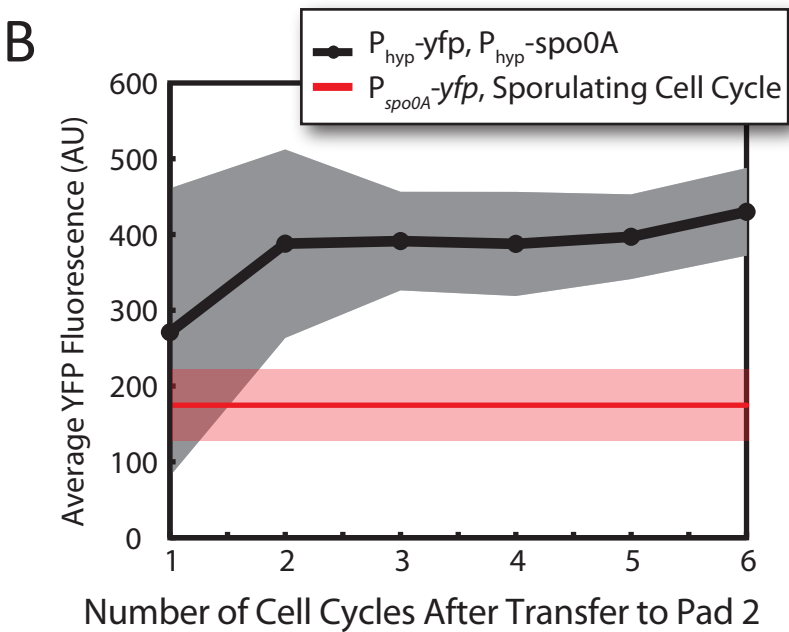

Supplement: Figure S2 — Analysis of pre-growth and Spo0A expression for pad transfer experiment (Figure 2E–G). (A) Distribution of number of cell divisions on Pad 1. The growth of 10 randomly chosen microcolonies were followed on Pad 1 using time lapse microscopy. (B) Induced Spo0A expression on Pad 2 (JL190, black—mean plus/minus SD) rapidly exceeds that from the wild type spo0A promoter (JL251, red—mean plus/minus SD). Fluorescence is mean cellular yfp intensity time averaged over the entire cell cycle. (PDF) [file pbio.1001252.s002.pdf]

Figure S3

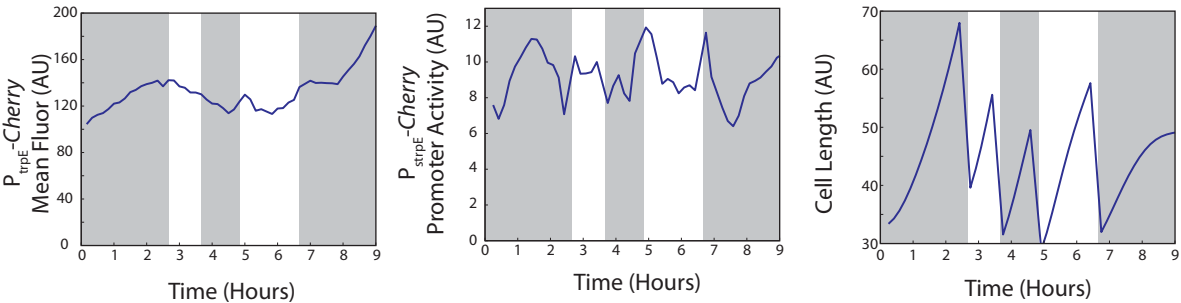

Supplement: Figure S3 — The trpE promoter fluctuates but does not pulse. Typical time traces of PtrpE-mCherry mean fluorescence (left) and promoter activity (center), along with cell length (right) in a typical cell lineage (strain JL024). Promoter activity, while fluctuating, has a lower dynamic range and less temporal structure than Spo0AP regulated promoters. (PDF) [file pbio.1001252.s003.pdf]

Figure S4

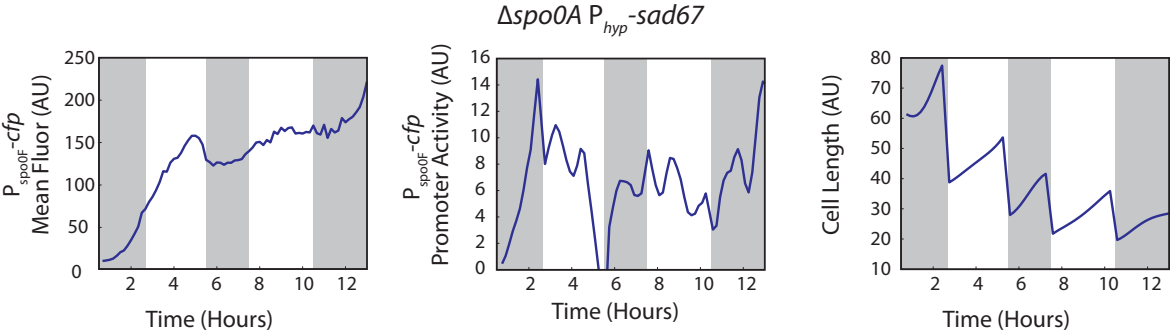

Supplement: Figure S4 — Pulsing is abolished in the constitutively active Spo0A mutant Spo0Asad67. Typical time traces of Pspo0F-yfp mean fluorescence (left) and promoter activity (center), along with cell length (right) in a typical cell lineage (strain JL065). The promoter activity exhibited fluctuations but lacked the characteristic cell cycle phased pulses present in the wild type. (PDF) [file pbio.1001252.s004.pdf]

Figure S5

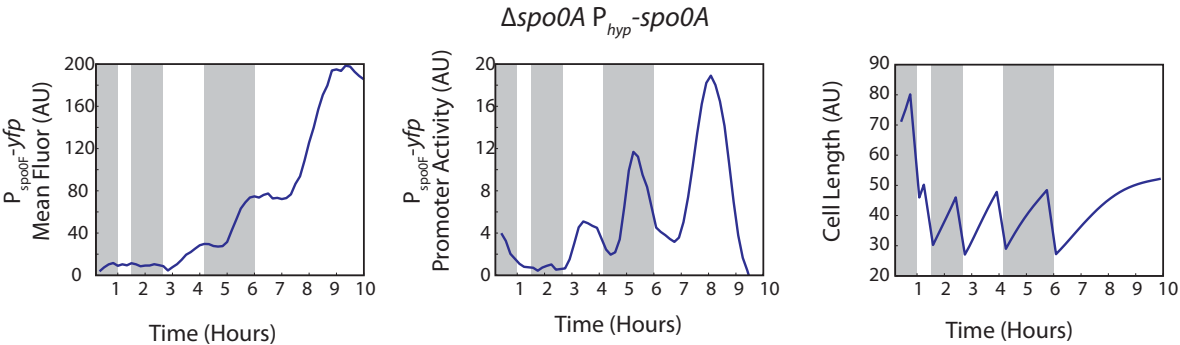

Supplement: Figure S5 — The native spo0A promoter is not required for pulsing. Typical time traces of Pspo0F-yfp mean fluorescence (left) and promoter activity (center), along with cell length (right) in a typical cell lineage of strain JL111 (Δspo0A Phyperspank-spo0A), showing pulsing similar to that observed in wild type cells. (PDF) [file pbio.1001252.s005.pdf]

Figure S6

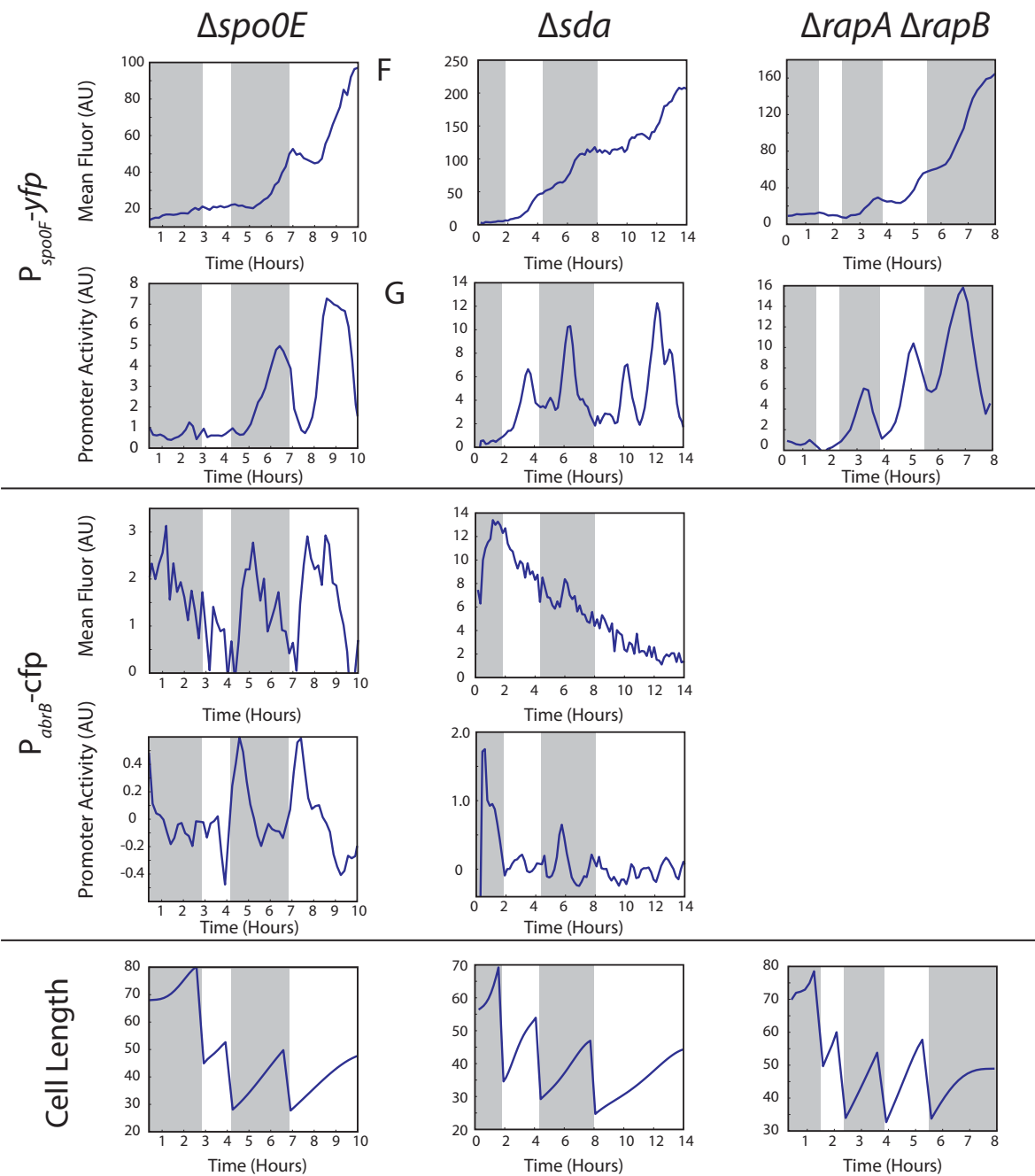

Supplement: Figure S6 — Negative regulators of sporulation initiation are not required for pulsing. Typical time traces of Pspo0F-yfp mean fluorescence (first row) and promoter activity (second row), PabrB-cfp mean fluorescence (third row) and promoter activity (fourth row), along with cell length (bottom row) in typical cell lineages of Dspo0E (strain JL014), Δsda (strain JL015), and ΔrapA ΔrapB (strain JL160). JL160 lacks the PabrB-cfp reporter present in the two other starins. Each strain exhibits Spo0AP activity pulses in the Pspo0F promoter similar to those seen in the wild type. (PDF) [file pbio.1001252.s006.pdf]

Figure S7

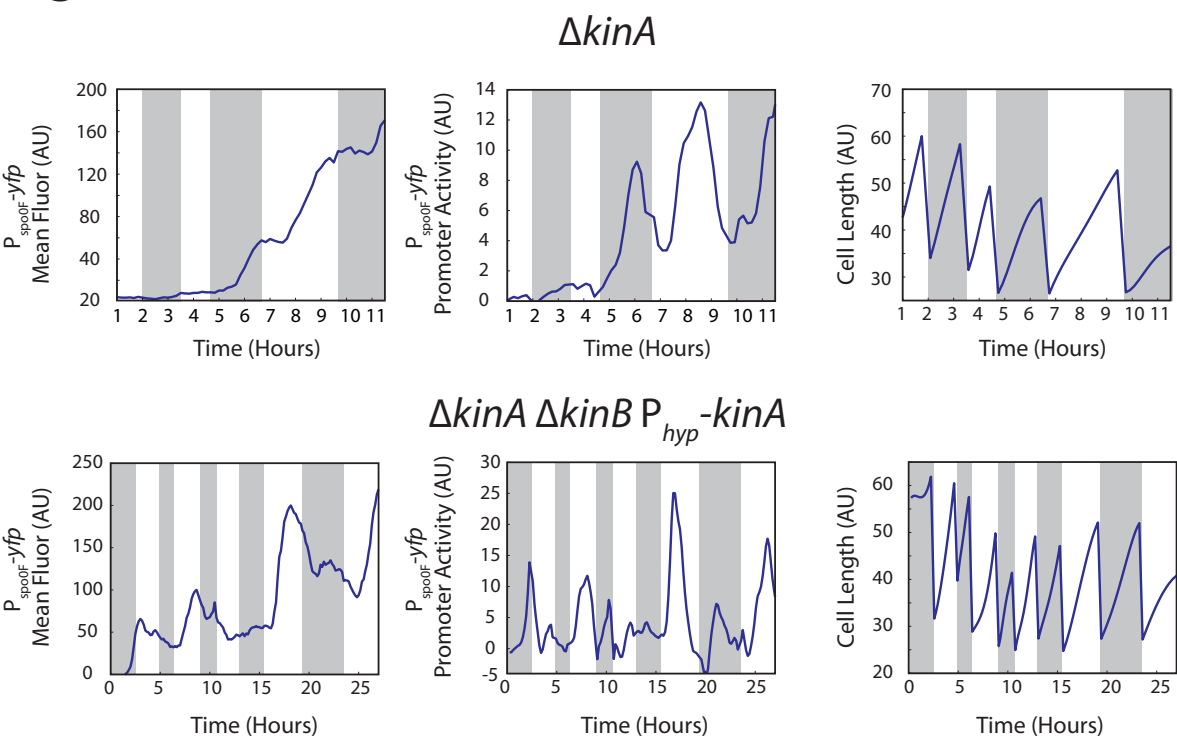

Supplement: Figure S7 — Major sporulation kinases are not individually required for pulsing. Typical time traces of Pspo0F-yfp mean fluorescence (left) and promoter activity (center), along with cell length (right) in typical cell lineages of ΔkinA (strain JL090, top) and ΔkinAΔkinB Phyperspank-kinA (strain JL144, induced at 2 µM IPTG). Both strains exhibit clear pulsing in Pspo0F-yfp promoter activity. (PDF) [file pbio.1001252.s007.pdf]

Figure S9

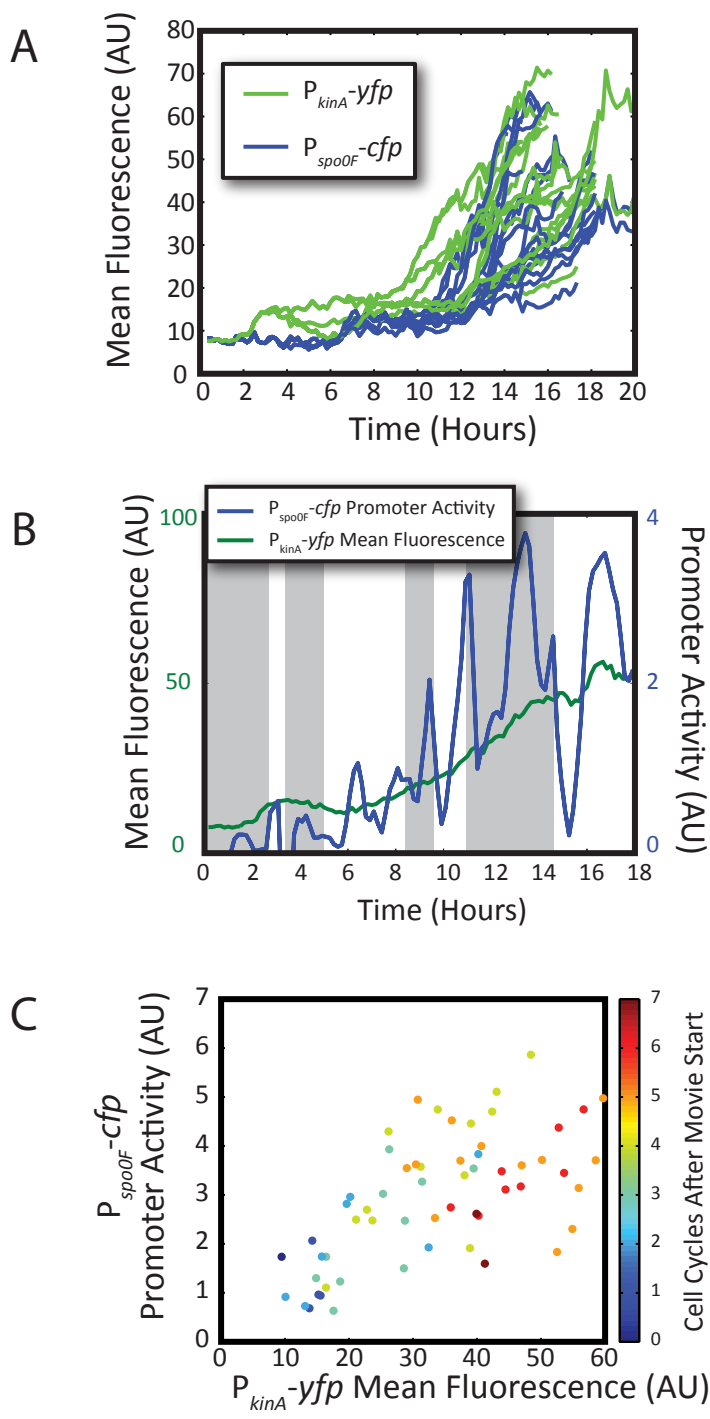

Supplement: Figure S9 — KinA levels increase gradually throughout the deferral period along with Spo0AP pulse amplitudes. (A) Mean cellular fluorescence traces of cells (strain JL264) expressing PkinA-yfp and Pspo0F-cfp. (B) PkinA-yfp mean fluorescence and Pspo0F-cfp promoter activity in a single cell lineage. Alternating grey and white shading represents successive cell divisions. (C) PkinA-yfp mean cellular fluorescence correlates with Spo0AP pulse amplitude. Each point represents a single cell's time averaged mean cellular yfp fluorescence (x-axis) and its maximum Pspo0F-cfp promoter activity (y-axis). Point color represents that cell's depth in the lineage tree (cell cycles). Correlation coefficient R = 0.68. (PDF) [file pbio.1001252.s009.pdf]

Figure S10

A

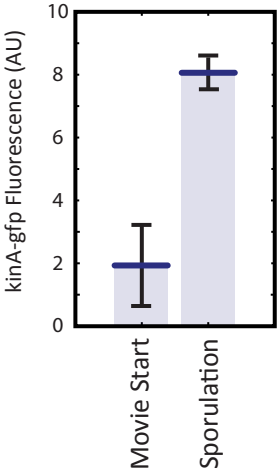

B

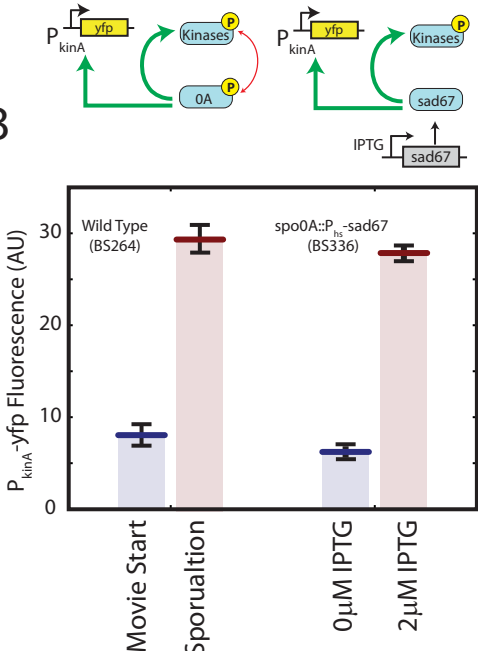

Supplement: Figure S10 — Regulation of Kinase Production. (A) kinA-gfp levels increase during sporulation. Mean cellular gfp fluorescence at movie start (left) and at sporulation (right). (B) Comparison of PkinA-yfp expression (mean cellular fluorescence) between cells with wild-type promoter regulation (left, BS264) and cells where PkinA is regulated by inducible spo0Asad67 (right, BS336). In wild type cells expression rises monotonically from movie start (blue) until sporulation (red). Fluorescence values in the inducible sad67 cell line were taken after 15 h on the resuspension media pad. Fluorescence in uninduced cells (blue) remained low, while the fluorescence of IPTG induced cells (red) was similar to that of sporulating wild type cells. Bars represent standard error of measurement. (PDF) [file pbio.1001252.s010.pdf]

Figure S11

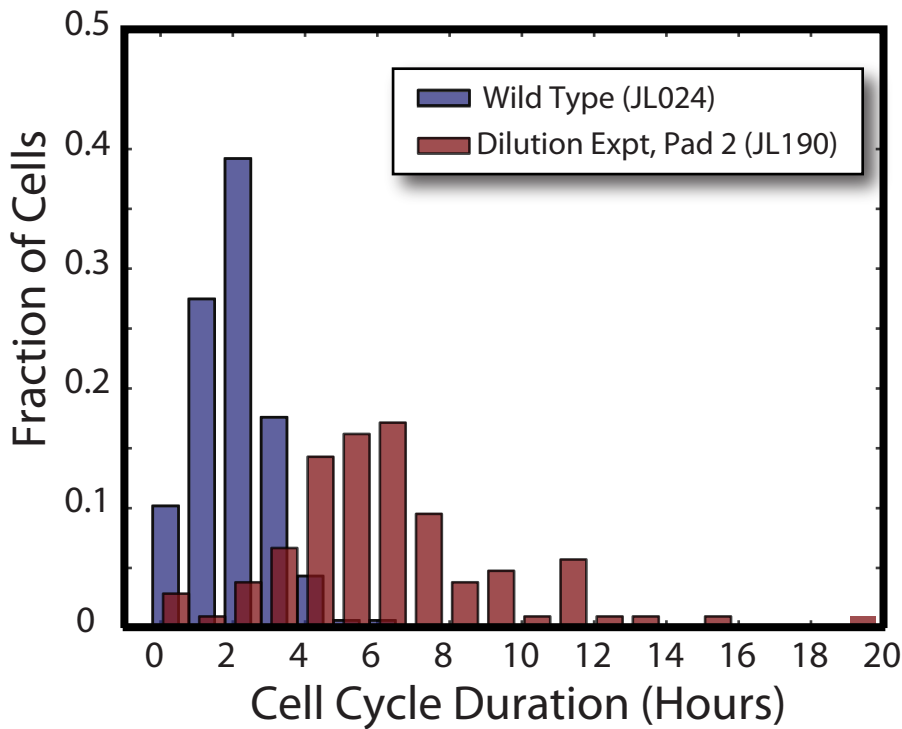

Supplement: Figure S11 — Cell growth rate in pad transfer experiments. Histograms of cell growth rate, measured in cell cycle duration on pads 1 and 2 (cf. Figure 2E–G). Growth on Pad 2 (red, N = 167) is significantly slower than growth on pad 1 (blue, N = 105). Although Pad 2 cells grow significantly slower than wild type cells, they still defer sporulation for the same number of cell cycles (Figure 2G). (PDF) [file pbio.1001252.s011.pdf]

Figure S12

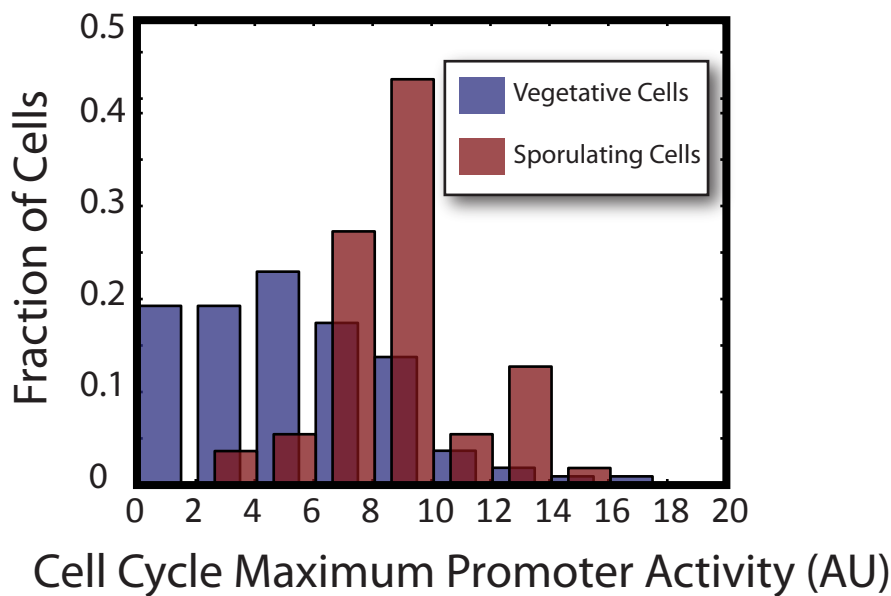

Supplement: Figure S12 — Sporulation occurs past a threshold level of Spo0AP. Histogram of maximal promoter activities (strain JL024) in our movie conditions. Non-sporulating cells (blue, N = 109) showed systematically lower promoter activity than sporulating cells (red, N = 55), although there is significant overlap. The conditional probability of sporulating given a promoter activity greater than 6 is 70%. (PDF) [file pbio.1001252.s012.pdf]

# Figure S13

## A

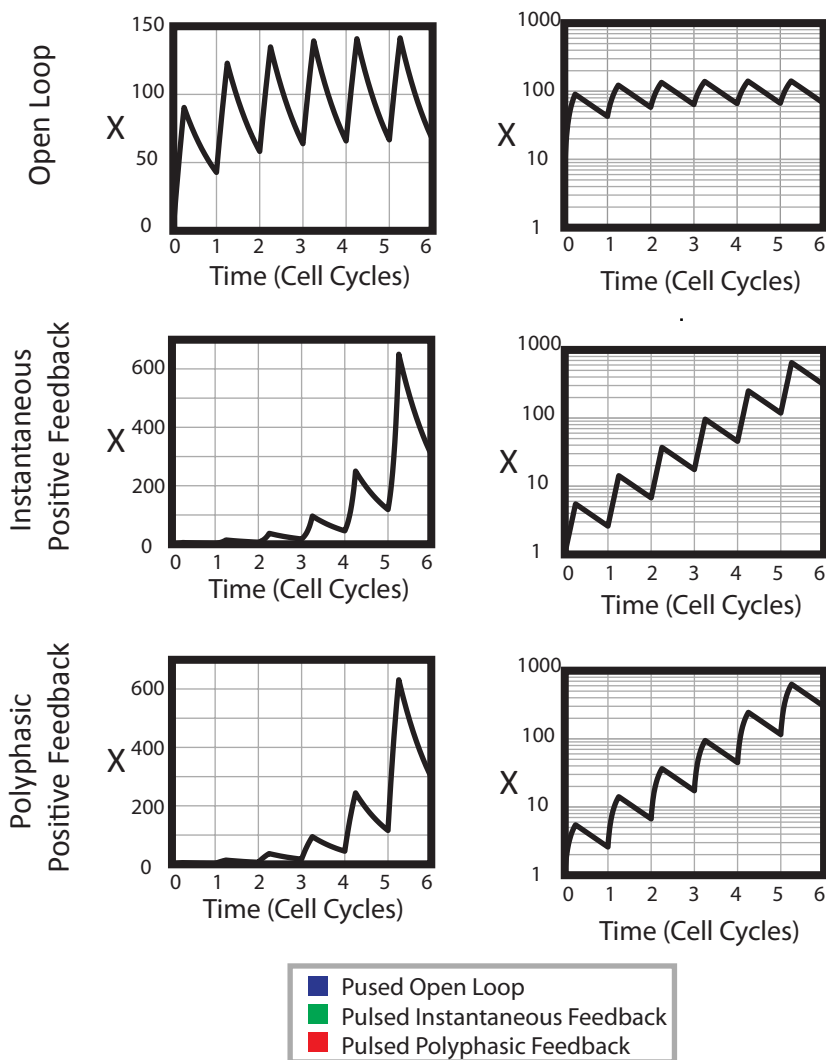

## B

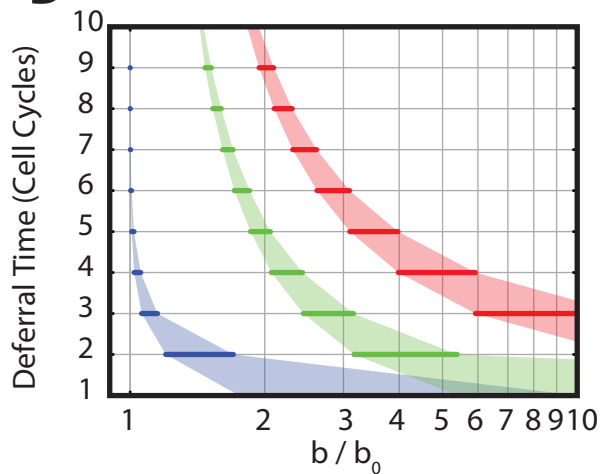

## C

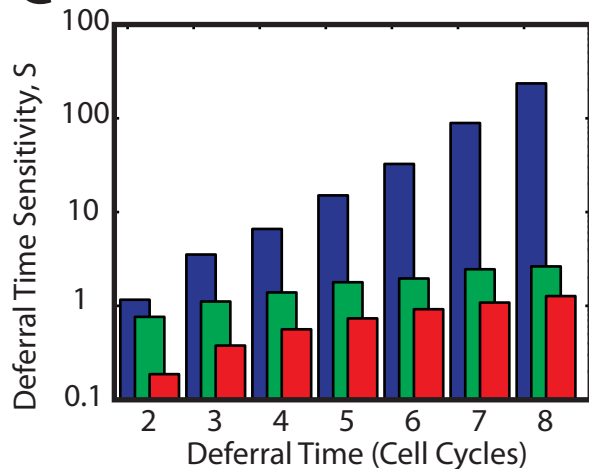

Supplement: Figure S13 — Simplified one-dimensional models capture qualitative circuit deferral behaviors. One-dimensional models are described in Text S1. (A) Dynamic traces of models tuned to cross a threshold of X = 100, starting from X = 1, with a five cell cycle deferral. X is plotted on both linear (left) and logarithmic (right) scales to illustrate exponential behavior. (B) Deferral time dependence on feedback strength for each model. For comparison, b of each model is plotted normalized to the minimal value b0 needed to reach the threshold of X = 100. Open loop: b0 = 100; instantaneous: b0 = 1; polyphasic: b0 = e−1. (C) One-dimensional models were compared for their ability to generate multi-cell-cycle deferral times, as with the two-component model of the main text. For each circuit, feedback strength b was tuned to produce different deferral times (x-axis). The sensitivity of deferral time to feedback strength was calculated as in the two-component model. The three circuits differ systematically in both the magnitude and rate of increase of sensitivity with deferral time. The open loop circuit is the most sensitive, followed by the instantaneous feedback, with the polyphasic feedback showing the least sensitivity. (PDF) [file pbio.1001252.s013.pdf]
