# Supplementary material for: Pulsed Feedback Defers Cellular Differentiation
Source: PLoS Biol. 2012 Jan 31;10(1):e1001252. doi: 10.1371/journal.pbio.1001252 (PMC3269414; doi:10.1371/journal.pbio.1001252)
Supplement: Figure S8 — Negative regulators of sporulation initiation are not required for a multi-cell-cycle deferral. For each strain (left column), 10 sporulating colonies were tracked with time lapse microscopy and mean sporulation time in cell cycles quantified using the T50 statistic (right column). Despite some day-to-day variation, no strain ever exhibited mean sporulation time less than three cell cycles. (PDF) [file pbio.1001252.s008.pdf]

Figure S8

Genotype	Cell Cycles (Mean / Std Error)
Δspo0E ΔrapA ΔrapB (JL287)	3.5 / 0.1
ΔrapA ΔrapB (JL184)	3.2/ 0.2
Δspo0E ΔynzD Δyisl (JL289)	3.3/ 0.2
ΔcodY (JL230)	4.5 / 0.2
ΔrapE (JL260)	4.3 / 0.2
ΔrapH (JL296)	4.2 / 0.1
ΔrapJ (JL300)	4.6 / 0.2
