# Supplementary material for: Pulsed Feedback Defers Cellular Differentiation
Source: PLoS Biol. 2012 Jan 31;10(1):e1001252. doi: 10.1371/journal.pbio.1001252 (PMC3269414; doi:10.1371/journal.pbio.1001252)
Supplement: Text S1 — Details of experimental protocols (including strain construction) and mathematical modeling. (DOC) [file pbio.1001252.s014.doc]

**Pulsed Feedback Defers Cellular Differentiation**

**Supplementary Information**

1. Simple Analytic Model of Pulsed Positive Feedback Dynamics
2. Computational Model of Spo0A Pulsed Positive Feedback Circuit Dynamics
3. Supporting References

**1. Simple Analytic Model of Pulsed Positive Feedback Dynamics.**

We explore here how pulsing affects the ability of positive feedback loops to generate and control extended deferrals. We first consider a simple set of one-dimensional dynamical systems, which use positive feedback to grow exponentially with time. These systems lack saturation but nevertheless provide qualitative insights into the effects observed in more realistic models. We will analyze the time it takes for each system to start from an initial low level of activation and grow to cross a higher threshold level, and how this “rise time” depends on the feedback strength in the system.

Each of these models considers a single protein concentration $x(t)$ which responds to two opposing effects: first order decay with time constant $\gamma$ modeling protein dilution from cell growth, and a positive feedback term $f(x)$which serves to increase $x(t)$. The combination of these two mechanisms provides a highly simplified phenomenological model for how a protein’s concentration feeds back to regulate its own transcription. The system’s dynamics are thus (dot denotes time derivative):

Within this framework, we will consider different forms of positive feedback, $f(x)$, and how they affect the rise time, defined as the time required for $x(t)$ to grow from $x_{0}$ to a threshold value $x_{F}$. This analysis will demonstrate how different pulsed positive feedback schemes affect the growth dynamics of $x(t)$, and therefore, the rise time.

**Constitutive Production**

We first consider a model lacking feedback, where the production of $x(t)$ is constant, e.g$f(x)=\beta$. This model could represent the case where kinase is controlled by a constitutive promoter. In this case $x(t)$ grows towards a steady state value $\beta/\gamma$ with time constant $\gamma$set by the cell cycle.

$$x(t)=\frac{\beta}{\gamma}\left(1-e^{-\gamma t}\right)+x_{0}e^{-\gamma t}$$

If $\frac{\beta}{\gamma}>x_{F}$ the system will eventually cross the threshold; if not, it will never do so. The curves in Fig. 5A are solutions to this model. The case with pulsed constitutive production behaves similarly (Figure S13A, left column), with the average value of $x(t)$ converging to steady state in about one cell cycle.

**Positive Feedback**

We next consider a model where $x(t)$ feeds back to activate its own production, e.g. $f(x)=\beta x$, representing simple linear positive feedback. In this case $x(t)$ grows exponentially with rate $\beta-\gamma$:

$$x(t)=x_{0}e^{(\beta-\gamma)t}$$

If the positive feedback strength is greater than the dilution rate $(\beta>\gamma)$, $x(t)$ will eventually exceed the threshold; if not, it will either dilute away or, in the marginal case $(\beta=\gamma)$remain unchanged.

**Pulsing**

What happens if the production occurs in pulses, as it does in the *B. subtilis* sporulation initiation circuit? For simplicity we consider square pulses of unit area:

$$U_{\Delta t}(t)=\frac{1}{\Delta t},0\leq t\leq\Delta t$$

$$U_{\Delta t}(t)=0,\Delta t<t\leq T$$

The pulses occur periodically with period $T$. Since Spo0A pulses occur approximately once per cell cycle, $T$ would be the cell cycle time.

**Pulsed Constitutive Production**

We consider a network where constitutive production occurs in pulses (Figure S13A). This is analogous to the network considered in Figure 6A.

Here, the time averaged value of $x(t)$relaxes to a steady state in about one cell cycle, as in the non-pulsed case.

**Pulsed Positive Feedback**

The dynamics in this case become:

It is convenient to solve this model one period at a time, e.g. for $0\leq t<T$, $T\leq t<2T$, etc. Integrating through the first cell cycle yields

$$x(T)=x_{0}e^{\beta}e^{-\gamma T}$$

After $N$ cell cycles

$$x(NT)=x_{0}e^{\beta N}e^{-\gamma NT}$$

Thus, in this model, from period to period, $x(t)$ grows exponentially with a growth rate constant, $\beta-\gamma T$. Note that this growth rate depends linearly on the feedback strength$\beta$.

**Polyphasic Positive Feedback­**

Finally, we consider a second type of feedback model where $x(t)$ activates its own production in pulses. In this model, although each pulse produces addition $x(t)$, it cannot feed back in time to amplify $x(t)$ production during that particular pulse. This case is analogous to the model in Figure 6C, where kinase production caused by a Spo0A^P^ pulse is delayed until after the pulse finishes. Thus, the production rate of $x(t)$ during each pulse is set by the amount of $x(t)$ at the start of the pulse. The full dynamics are:

Integrating through one period, as before, and taking the short pulse limit as $\Delta t\to0$,

$$\lim_{\Delta t\to0} x(T)=(1+\beta)e^{-\gamma T}$$

After $N$ cell cycles

$$x(NT)=e^{\log(1+\beta)^{N}}e^{-\gamma NT}$$

In this model, from period to period, $x(t)$ grows exponentially with a rate constant of $\log(1+\beta)-\gamma T$. Note that this growth rate depends logarithmically on the feedback strength$\beta$, instead of linearly in the instantaneous positive feedback case. Identical scaling is obtained if the feedback is taken to be a Dirac $\delta$ function, or if we account for dilution of $x(nT)$during each pulse.

**Model Comparisons**

How do the models compare in their ability to set deferral times (Figure S13B,C)? For each model, we calculated each deferral time’s sensitivity to changes in feedback strength. We defined deferral time sensitivity as $S=\partial\log T_{D}/\partial\log\beta$, as in Figure 6E. In the open loop model, deferral times longer than 1 cell cycle were extremely sensitive to changes in $\beta$(blue bars). In the instantaneous positive feedback model (green bars), deferral times were significantly less sensitive to $\beta$ because positive feedback competes with protein dilution to set the growth rate of $x(t)$. Finally, deferral times in the polyphasic positive feedback model were even less sensitive to feedback strength (red bars). This extra reduction in sensitivity arises because production of $x(t)$ during each pulse is constant, rather than continuously compounding as in the instantaneous feedback case.

**2. Computational Model of Spo0A Network Dynamics**

To analyze the effects of pulsing and indirect feedback on the accumulation of Spo0A activity, we considered a set of dynamical models corresponding to the cartoons in Fig. 6. The models consist of a sporulation histidine kinase ($K$) which phosphorylates a response regulator representing Spo0A ($A$). Phosphorylated Spo0A ($A^{P}$) feeds back to activate its own transcription directly and to activate transcription of $K$through an intermediate regulator$D$. A constant phosphatase activity ($k_{P}$) analgous to Spo0E drains phosphates directly from the response regulator. To reproduce the pulsing in Spo0A phosphorylation we modeled the autophosphorylation from the kinase to Spo0A as gated by a square wave$s(t)$, which takes the value 0 except during the third quarter of each cell cycle where it takes the value 1.

Model reactions, and corresponding rate constants, are summarized as follows, with $\varphi$ denoting degradation of the corresponding molecule:

| Table 1. List of reactions | |
| --- | --- |
| **Reaction** | **Description** |
|  | kinase autophosphorylation |
|  | kinase autodephosphorylation |
|  | forward phosphotransfer |
|  | reverse phosphotransfer |
|  | dephosphorylation by phosphatase |
|  | degradation/dilution of unphosphorylated kinase |
|  | degradation/dilution of unphosphorylated Spo0A |
|  | degradation/dilution of phosphorylated Spo0A |
|  | degradation/dilution and dilution of D |
|  | production of unphosphorylated Spo0A |
|  | production of D |
|  | production of unphosphorylated kinase |

We modeled the transcription of $A$ and $K$ using Hill functions$f_{A}(A^{P})$ and $f_{K}(D)$ respectively. We assume that wild type promoters are activated by their cognate transcription factor. In this case $f_{A}=f_{A}(A^{P})=\beta_{A}\frac{A^{P}}{K_{D}+A^{P}}$, and $f_{K}=f_{K}(D)=\beta_{K}\frac{D}{K_{D}+D}$. For simplicity we assume the promoter of the intermediate delay element is linear, $f_{D}=f_{D}(A^{P})=\beta_{D}A^{P}$.

We modeled these reactions as ordinary differential equations for the concentrations of the four reactants$K,K^{P},A,A^{P}$, as well as $D$. Equations were numerically integrated in Matlab using the built in ode15s solver. The equations are:

$$\frac{dK}{dt}=-\gamma K+f_{K}(D)-\alpha_{A}s(t)K+\alpha_{D}K^{P}+k_{F}K^{P}A-k_{R}KA^{P}$$

$$\frac{dK^{P}}{dt}=-\gamma K^{P}+\alpha_{A}s(t)K-\alpha_{D}K^{P}-k_{F}K^{P}A+k_{R}KA^{P}$$

$$\frac{dD}{dt}=-\gamma_{D}D+f_{D}(A^{P})$$

$$\frac{dA}{dt}=-\gamma A+f_{A}(A^{P})-k_{F}K^{P}A+k_{R}KA^{P}+k_{P}A^{P}$$

$$\frac{dA^{P}}{dt}=-\gamma A^{P}+k_{F}K^{P}A-k_{R}KA^{P}-k_{P}A^{P}$$

The choice of kinetic parameters was guided by previously published biochemical results, along with our own data. The protein decay constant $\gamma$ was chosen to match the average cell cycle time of 2 hrs in our experiments, since none of the proteins we model are known to be unstable. Kinase autophosphorylation $\alpha_{A}$was chosen as linear since the likely large molar excess of cellular ATP over kinase allows the reaction to be approximated as a Michaelis-Menten reaction in the linear regime. The order of magnitude of the autophosphorylation constant is consistent with previously determined reaction rates. The kinase autodephosphorylation rate $\alpha_{D}$was chosen to match the previously measured dephosphorylation rate of KinA . Forward and reverse phosphotransfer rates were set large enough to not be rate limiting and equal to each other allow reversible phosphotransfer.

Spo0A promoter strength was set to ensure approximately 10,000 Spo0A molecules are present when cells reach the sporulation threshold, as determined in . As expression from *P_kinA_* and *P_kinB_* promoter fusions was consistently weaker than expression from *P_spo0A_* in our conditions, the kinase promoter strength range was set to a value 10 - 100 times weaker than *spo0A* promoter strength. The binding constant of activators to these promoters was chosen as 100 molecules per cell, which is greater than the 64nm binding constant of the high affinity *spo0A* repressed promoter *abrB* but lower than that of many Stage II sporulation genes.

Sporulation was defined to have occurred when the cell cycle averaged amount of Spo0A exceeded 200 molecules per cell. We chose this threshold as similar to the binding constant of many Stage II sporulation genes.

For the polyphasic positive feedback model, the dilution rate $\gamma_{D}$of the intermediate $D$ was set equal to that of all other proteins. For the instantaneous positive feedback model $\gamma_{D}$ was set to be 100 fold greater than the dilution rate. To maintain equivalent steady state $D$ levels, we set$\beta_{D}$ in the instantaneous model to be 100 times that in the polyphasic feedback model.

We tuned our circuits to facilitate a controlled comparison of rise times between instantaneous positive feedback and polyphasic positive feedback models. We chose parameters so that for a given $\beta_{K}$, steady state cell cycle averaged $A^{P}$ concentrations in the polyphasic model was greater than or equal to those of the instantaneous model. This ensures that at a given $\beta_{K}$ the slower deferral in the polyphasic model is due solely to slower protein accumulation, and not to a lower steady state.

| Table 2**: Circuit parameter values for deferral sensitivity simulations of Figure 6D.** | | | |
| --- | --- | --- | --- |
| **Parameter (units)** | **Open Loop** | **Instantaneous Positive Feedback** | **Polyphasic Positive Feedback** |
| $\alpha_{A}$ (1/hr) | 1500 | 1500 | 2200 |
| $\alpha_{D}$ (1/hr) | 5 | 5 | 5 |
| $k_{F}$ (1/hr-molecules) | 1000 | 1000 | 1000 |
| $k_{R}$ (1/hr-molecules) | 1000 | 1000 | 1000 |
| $k_{P}$ (1/hr) | 50 | 50 | 60 |
| $\gamma$ (1/hr) | 0.35 | 0.35 | 0.35 |
| $\gamma$ (1/hr) | NA | 35 | 0.35 |
| $\beta_{A}$ (molecules/hr) | 10,000 | 10,000 | 10,000 |
| $\beta_{D}$ (molecules/hr) | NA | 3.5 | 0.035 |
| $\beta_{K}$ | Varied | Varied | Varied |
| $K_{D}$ (molecules) | 100 | 100 | 100 |

**3. Methods**

**Plasmids and Strain Construction**

All *B. subtilis* strains were constructed in the PY79 genetic background. Plasmids were constructed using standard molecular cloning techniques in *E. coli* DH5α. Full sequences are available by request. Unless otherwise noted, plasmid constructs were integrated into indicated chromosomal regions via double crossover using standard *B. subtilis* transformation protocols.

All plasmids beginning with the designation “ECE” were obtained from the Bacillus Genetic Stock Center (BGSC) at Ohio State University in Columbus, Ohio USA (<http://www.bgsc.org/>).

*Transcriptional Reporters*

Fluorescent transcriptional reporters were cloned into the plasmids pDL30 (BamHI/EcoRI cloning site, *amyE* locus integration), ECE174 (BamHI/EcoRI cloning site, *sacA* locus integration), or pVK317 (ClaI/KpnI cloning site, *ppsB* locus integration). Briefly, PCR was used to amplify approximately 500 base pairs of genomic DNA upstream of the relevant gene coding sequence up to the start codon. The amplified product was then fused using PCR to a fluorescent reporter gene. Primers for the fusion reaction contained flanking terminators to minimize read-through and appropriate restriction sites for cloning into integration vectors. All plasmid constructs were sequenced for correctness before integration.

*Chromosomal Deletions*

Chromosomal deletion strains were obtained from cited sources, and the reference given when available. Relevant deletions were moved to our wild type strain background by transforming genomic DNA and the integrations were checked by PCR.

*Spo0A Deletion*

Approximately 500bp regions upstream and downstream of the Spo0A ORF were cloned into the ECE171 plasmid for double crossover integration. The upstream region was cloned in using MfeI/SacI, while the downstream region was cloned in using NarI/SalI.

*Overexpression Constructs*

Overexpression constructs (Spo0A, and Spo0F-Spo0B-Spo0A operon) were made by cloning the appropriate coding sequences into the pDR111 expression vector, for expression from the IPTG inducible hyperspank promoter. The Spo0F coding sequence was taken using only the second of two consecutive ATG start codons. Each individual coding sequence in the operon was separated by a spacing region and an optimized ribosome binding site.

| **Table** 3**: Strains Used in This Study** | | | |
| --- | --- | --- | --- |
| **Strain Name** | **Parent Strain** | **Integrated Construct** | **Genotype** |
| JL011 | PY79 | ppsB::P*_trpE_*-mCherry ErmR | ppsB::P*_trpE_*-mCherry ErmR |
| JL012 | JL011 | sacA::P*_abrB_*-CFP CmR | sacA::P*_abrB_*-CFP CmR, ppsB::P*_trpE_*-mCherry ErmR |
| JL013 | JL012 | amyE::P*_spo0F_*-YFP SpectR | amyE::P*_spo0F_*-YFP SpectR, sacA::P*_abrB_*-CFP CmR, ppsB::P*_trpE_*-mCherry ErmR |
| JL014 | JL013 | spo0E::TetR (BAL378) | Spo0E::TetR, amyE::P*_spo0F_*-YFP SpectR, sacA::P*_abrB_*-CFP CmR, ppsB::P*_trpE_*-mCherry ErmR |
| JL015 | JL013 | sda::KanR (SB321) | sda::KanR, amyE::P*_spo0F_*-YFP SpectR, sacA::P*_abrB_*-CFP CmR, ppsB::P*_trpE_*-mCherry ErmR |
| JL024 | JL011 | amyE::P*_spo0F_*-YFP SpectR | amyE::P*_spo0F_*-YFP SpectR, ppsB::P*_trpE_*-mCherry ErmR |
| JL028 | JL024 | SpectR::CmR (pDAG32) | amyE::P*_spo0F_*-YFP CmR, ppsB::P*_trpE_*-mCherry ErmR |
| JL034 | JL011 | rapB::SpectR (BAL366) | rapB::SpectR, ppsB::P*_trpE_*-mCherry ErmR |
| JL060 | JL011 | sacA::P*_sdp_*-CFP CmR | sacA::P*_sdp_*-CFP CmR, ppsB::P*_trpE_*-mCherry ErmR |
| JL064 | JL034 | rapA::CmR (BAL375) | rapA::CmR, rapB::SpectR, ppsB::P*_trpE_*-mCherry ErmR |
| JL065 | NA | Lab stock | Spo0A::P*_hyperspank_*-sad67 CmR, amyE::P*_spo0F_*-cfp PspoIIG-yfp NeoR, ppsB::P*_trpE_*-mCherry ErmR |
| JL072 | JL60 | amyE::P*_spo0F_*-YFP SpectR | amyE::P*_spo0F_*-YFP SpectR, sacA::P*_sdp_*-CFP CmR, ppsB::P*_trpE_*-mCherry ErmR |
| JL089 | JL024 | ErmR::NeoR (ECE77) | amyE∷P*_spo0F_*-yfp Spect R, ppsB∷P*_trpE_*-Cherry NeoR |
| JL090 | JL089 | kinA::ErmR | kinA::ErmR, amyE∷P*_spo0F_*-yfp SpectR, ppsB∷P*_trpE_*-Cherry NeoR |
| JL097 | JL16 | amyE::P*_spo0F_*-cfp SpectR | amyE::P*_spo0F_*-cfp SpectR, ppsB::P*_trpE_*-mCherry ErmR |
| JL101 | JL028 | CmR::NeoR (ECE73) | amyE::P*_spo0F_*-YFP NeoR, ppsB::P*_trpE_*-mCherry ErmR |
| JL106 | JL024 | spo0A::KanR | spo0A::kanR, amyE∷P*_spo0F_*-yfp SpectR, ppsB∷P*_trpE_*-Cherry ErmR |
| JL108 | JL024 | sacA::P*_hyperspank_*-spo0A CmR | sacA::P*_hyperspank_*-spo0A CmR, amyE::P*_spo0F_*-YFP, ppsB::P*_trpE_*-mCherry |
| JL110 | JL024 | sacA::P*_hyperspank_*-spo0A-spo0B-spo0F CmR | sacA::P*_hyperspank_*-spo0A-spo0B-spo0F CmR, amyE::P*_spo0F_*-YFP, ppsB::P*_trpE_*-mCherry |
| JL111 | JL108 | Spo0A::KanR | sacA::P*_hyperspank_*-spo0A CmR, spo0A::KanR, amyE::P*_spo0F_*-YFP, ppsB::P*_trpE_*-mCherry |
| JL113 | JL110 | Spo0A::KanR | sacA::P*_hyperspank_*-spo0A-spo0B-spo0F CmR, spo0A::KanR, amyE::P*_spo0F_*-YFP, ppsB::P*_trpE_*-mCherry |
| JL115 | PY79 | amyE::P*_spo0F_*-YFP SpectR | amyE::P*_spo0F_*-YFP SpectR |
| JL119 | JL101 | kinA::P*_hyperspank_*-kinA SpectR (MF1887) | kinA::P*_hyperspank_*-kinA SpectR, amyE::P*_spo0F_*-YFP NeoR, ppsB::P*_trpE_*-mCherry ErmR |
| JL131 | NA | Lab Stock | amyE::P*_hyperspank_*-YFP SpectR |
| JL160 | JL064 | amyE::P*_spo0F_*-YFP NeoR | amyE::P*_spo0F_*-yfp NeoR, rapA::CmR, rapB::SpectR, ppsB::P*_trpE_*-mCherry ErmR |
| JL164 | JL011 | kinC::KanR (JQ220) | kinC::KanR, ppsB::P*_trpE_*-mCherry ErmR |
| JL166 | JL164 | kinA::P*_hyperspank_*-kinA SpectR (MF1887) | kinA::P*_hyperspank_*-kinA SpectR, kinC::KanR, ppsB::P*_trpE_*-mCherry ErmR |
| JL167 | JL166 | kinB::TetR (JH19980) | kinA::P*_hyperspank_*-kinA SpectR, kinB::tetR, kinC::KanR, ppsB::P*_trpE_*-mCherry ErmR |
| JL168 | JL167 | amyE::P*_spo0F_*-YFP CmR | amyE::P*_spo0F_*-YFP CmR, kinA::P*_hyperspank_*-kinA SpectR, kinB::tetR, kinC::KanR, ppsB::P*_trpE_*-mCherry ErmR |
| JL172 | JL011 | ErmR::PhleoR (ECE78) | ppsB::P*_trpE_*-mCherry PhleoR |
| JL188 | JL011 | amyE::P*_hyperspank_*-yfp SpectR | amyE::P*_hyperspank_*-yfp SpectR, ppsB::P*_trpE_*-mCherry ErmR |
| JL189 | JL188 | sacA::P*_hyperspank_*-spo0A CmR | sacA::P*_hyperspank_*-spo0A CmR, amyE::P*_hyperspank_*-yfp SpectR, ppsB::P*_trpE_*-mCherry ErmR |
| JL190 | JL189 | Spo0A::KanR | Spo0A::KanR, sacA::P*_hyperspank_*-spo0A CmR, amyE::P*_hyperspank_*-yfp SpectR, ppsB::P*_trpE_*-mCherry ErmR |
| JL239 | MF929 | NA | kinA::kinA-gfp KanR |
| JL251 | JL172 | sacA::Pspo0A-yfp CmR | sacA::Pspo0A-yfp CmR, ppsB::P*_trpE_*-mCherry PhleoR |
| JL264 | JL97 | sacA::P*_kinA_*-yfp CmR | sacA::P*_kinA_*-yfp CmR, amyE::P*_spo0F_*-cfp SpectR, ppsB::P*_trpE_*-mCherry ErmR |
| JL335 | JL264 | CmR::TetR (ECE75) | sacA::P*_kinA_*-yfp TetR, amyE::P*_spo0F_*-cfp SpectR, ppsB::P*_trpE_*-mCherry ErmR |
| JL336 | JL335 | spo0A::P*_hyperspank_*-sad67 CmR | spo0A::P*_hyperspank_*-sad67 CmR, sacA::P*_kinA_*-yfp TetR, amyE::P*_spo0F_*-cfp SpectR, ppsB::P*_trpE_*-mCherry ErmR |

*Sources for Deletions*

Δsda: Moved from strain SB321, a kind gift from Steven Biller and William Burkholder (JL015)

Δspo0E: Moved from BAL378, a kind gift from Beth Lazazzera. (JL014)

ΔrapA: Moved from BAL375, a kind gift from Beth Lazazzera. (JL160)

ΔrapB: Moved from BAL366, a kind gift from Beth Lazazzera. (JL 160)

kinA∷P*_hyperspank_*-kinA: Moved from MF1887, a kind gift from M. Fujita and R. Losick. (JL119, JL144, JL168)

ΔkinB: Moved from JH19980, a kind gift from Jim Hoch. (JL144, JL168)

ΔkinC: Moved from JQ220, a kind gift from Alan Grossman. (JL168)

**3. Supplementary Figure Legends**

1. **Figure S1**: **Spo0A^P^ targets typically pulse once per cell cycle with defined phases**. (A) Single cell time lapse traces of promoter activity in P*_abrB_*-cfp/P*_spo0F_*-yfp cells (JL013, top) and P*_sdp_*-cfp/P*_spo0F_*-yfp cells (JL072, bottom). Individual cell cycles are delineated by sequential gray and white shading. Cartoon indicates key regulatory links. (B) Left: illustration of definition of phase. Right: Histogram of cell cycle phases for *abrB* and *spo0F* promoter activity pulses. *abrB* expression typically pulses early each cell cycle, while spo0F expression typically pulses later. (C) Histogram of number of spo0F promoter activity pulses per cell cycle. Half of cells pulse in the first two cell cycles following transfer to resuspension media, while the majority of cells show a single pulse in subsequent cell cycles. The null hypothesis is a binomial distribution with the same mean number of pulses per cell cycle.

2. **Figure S2: Analysis of pre-growth and Spo0A expression for pad transfer experiment (Fig. 2E-G)**. (A) Distribution of number of cell divisions on Pad 1. The growth of ten randomly chosen microcolonies were followed on Pad 1 using time lapse microscopy. (B) Induced Spo0A expression on Pad 2 (JL190, black - mean plus/minus std dev) rapidly exceeds that from the wild type *spo0A* promoter (JL251, red - mean plus/min std dev). Fluorescence is mean cellular yfp intensity time averaged over the entire cell cycle.

3. **Figure S3: The trpE promoter fluctuates but does not pulse.** Typical time traces of P*_trpE_*-mCherry mean fluorescence (left) and promoter activity (center), along with cell length (right) in a typical cell lineage (strain JL024). Promoter activity, while fluctuating, has a lower dynamic range and less temporal structure than Spo0A^P^ regulated promoters.

4. **Figure S4: Pulsing is abolished in the constitutively active Spo0A mutant Spo0A^sad67^.** Typical time traces of P*_spo0F_*-yfp mean fluorescence (left) and promoter activity (center), along with cell length (right) in a typical cell lineage (strain JL065). The promoter activity exhibited fluctuations but lacked the characteristic cell cycle phased pulses present in the wild type.

5. **Figure S5: The native spo0A promoter is not required for pulsing.** Typical time traces of P*_spo0F_*-yfp mean fluorescence (left) and promoter activity (center), along with cell length (right) in a typical cell lineage of strain JL111 (∆spo0A P_hyperspank_-spo0A), showing pulsing similar to that observed in wild type cells.

6. **Figure S6: Negative regulators of sporulation initiation are not required for pulsing.** Typical time traces of P*_spo0F_*-yfp mean fluorescence (first row) and promoter activity (second row), P*_abrB_*-cfp mean fluorescence (third row) and promoter activity (fourth row), along with cell length (bottom row) in typical cell lineages of Δspo0E (strain JL014), Δsda (strain JL015), and ΔrapA ΔrapB (strain JL160). JL160 lacks the P*_abrB_*-cfp reporter present in the two other starins. Each strain exhibits Spo0A^P^ activity pulses in the P*_spo0F_* promoter similar to those seen in the wild type.

7. **Figure S7: Major sporulation kinases are not individually required for pulsing.** Typical time traces of P*_spo0F_*-yfp mean fluorescence (left) and promoter activity (center), along with cell length (right) in typical cell lineages of ΔkinA (strain JL090, top), ΔkinAΔkinB P*_hyperspank_*-kinA (strain JL144, induced at 2µM IPTG). Both strains exhibit clear pulsing in P*_spo0F_*-yfp promoter activity.

8. **Figure S8: Negative regulators of sporulation initiation are not required for a multi-cell-cycle deferral.** For each strain (left column), 10 sporulating colonies were tracked with time lapse microscopy and mean sporulation time in cell cycles quantified using the T_50_ statistic (right column). Despite some day to day variation, no strain ever exhibited mean sporulation time less than 3 cell cycles.

9. **Figure S9: KinA levels increase gradually throughout the deferral period along with Spo0A^P^ pulse amplitudes.** (A) Mean cellular fluorescence traces of cells (strain JL264) expressing P*_kinA_*-yfp and P*_spo0F_*-cfp. (B) P*_kinA_*-yfp mean fluorescence and P*_spo0F_*-cfp promoter activity in a single cell lineage. Alternating grey and white shading represents successive cell divisions. (C) P*_kinA_*-yfp mean cellular fluorescence correlates with Spo0A^P^ pulse amplitude. Each point represents a single cell’s time averaged mean cellular yfp fluorescence (x-axis) and its maximum P*_spo0F_*-cfp promoter activity (y-axis). Point color represents that cell’s depth in the lineage tree (cell cycles). Correlation coefficient R = 0.68.

10. **Figure S10: Regulation of Kinase Production.** (A) kinA-gfp levels increase during sporulation. Mean cellular gfp fluorescence at movie start (left) and at sporulation (right). (B) Comparision of P*_kinA_*-yfp expression (mean cellular fluorescence) between cells with wild-type promoter regulation (left, BS264) and cells where P*_kinA_* is regulated by inducible spo0A^sad67^ (right, BS336). In wild type cells expression rises monotonically from movie start (blue) until sporulation (red). Fluorescence values in the inducible sad67 cell line were taken after 15 hours on the resuspension media pad. Fluorescence in uninduced cells (blue) remained low, while the fluorescence of IPTG induced cells (red) was similar to that of sporulating wild type cells. Bars represent standard error of measurement.

11. **Figure S11: Cell growth rate in pad transfer experiments.** Histograms of cell growth rate, measured in cell cycle duration on pads 1 and 2 (cf. Fig. 2E-G). Growth on Pad 2 (red, N=167) is significantly slower than growth on pad 1 (blue, N=105). Although Pad 2 cells grow significantly slower than wild type cells, they still defer sporulation for the same number of cell cycles (Fig. 2G).

12. **Figure S12: Sporulation occurs past a threshold level of Spo0A^P^.** Histogram of maximal promoter activities (strain JL024) in our movie conditions. Non-sporulating cells (blue, N=109) showed systematically lower promoter activity than sporulating cells (red, N=55), although there is significant overlap. The conditional probability of sporulating given a promoter activity greater than 6 is 70%.

13. **Figure S13: Simplified one-dimensional models capture qualitative circuit deferral behaviors.** One-dimensional models are described in supplementary material, section 1. (A) Dynamic traces of models tuned to cross a threshold of X=100, starting from X=1, with a 5 cell cycle deferral. X is plotted on both linear (left) and logarithmic (right) scales to illustrate exponential behavior. (B) Deferral time dependence on feedback strength for each model. For comparison, b of each model is plotted normalized to the minimal value b0 needed to reach the threshold of X=100. Open loop: b_0_ = 100; instantaneous: b_0_ = 1; polyphasic: b_0_ = e-1.(C) One dimensional models were compared for their ability to generate multi-cell cycle deferral times, as with the two component model of the main text. For each circuit, feedback strength b was tuned to produce different deferral times (x axis). The sensitivity of deferral time to feedback strength was calculated as in in the two component model. The three circuits differ systematically in both the magnitude and rate of increase of sensitivity with deferral time. The open loop circuit is the most sensitive, followed by the instantaneous feedback, with the polyphasic feedback showing the least sensitivity.

**4. Supplementary References**

1. Bennett, B.D., et al., *Absolute metabolite concentrations and implied enzyme active site occupancy in Escherichia coli.* Nat Chem Biol, 2009. **5**(8): p. 593-9.

2. Grimshaw, C.E., et al., *Synergistic kinetic interactions between components of the phosphorelay controlling sporulation in Bacillus subtilis.* Biochemistry, 1998. **37**(5): p. 1365-75.

3. Chastanet, A., et al., *Broadly heterogeneous activation of the master regulator for sporulation in Bacillus subtilis.* Proc Natl Acad Sci U S A, 2010. **107**(18): p. 8486-91.

4. Fujita, M., J.E. Gonzalez-Pastor, and R. Losick, *High- and low-threshold genes in the Spo0A regulon of Bacillus subtilis.* J Bacteriol, 2005. **187**(4): p. 1357-68.

5. Silvaggi, J.M., et al., *Unmasking novel sporulation genes in Bacillus subtilis.* J Bacteriol, 2004. **186**(23): p. 8089-95.

6. Fujita, M. and R. Losick, *Evidence that entry into sporulation in Bacillus subtilis is governed by a gradual increase in the level and activity of the master regulator Spo0A.* Genes Dev, 2005. **19**(18): p. 2236-44.

7. Wang, L., et al., *A novel histidine kinase inhibitor regulating development in Bacillus subtilis.* Genes Dev, 1997. **11**(19): p. 2569-79.

8. Quisel, J.D., W.F. Burkholder, and A.D. Grossman, *In vivo effects of sporulation kinases on mutant Spo0A proteins in Bacillus subtilis.* J Bacteriol, 2001. **183**(22): p. 6573-8.
